# Colorectal Cancer Stage at Diagnosis Before vs During the COVID-19 Pandemic in Italy

**DOI:** 10.1001/jamanetworkopen.2022.43119

**Published:** 2022-11-21

**Authors:** Matteo Rottoli, Alice Gori, Gianluca Pellino, Maria Elena Flacco, Cecilia Martellucci, Antonino Spinelli, Gilberto Poggioli

**Affiliations:** 1 Surgery of the Alimentary Tract, IRCCS Azienda Ospedaliero–Universitaria di Bologna, Bologna, Italy; 2 Department of Medical and Surgical Sciences, Alma Mater Studiorum University of Bologna, Bologna, Italy; 3 Department of Advanced Medical and Surgical Sciences, Università degli Studi della Campania Luigi Vanvitelli, Naples, Italy; 4 Colorectal Surgery, University Hospital Vall d’Hebron, Barcelona, Spain; 5 Department of Environmental and Preventive Sciences, University of Ferrara, Ferrara, Italy; 6 Department of Biomedical Sciences, Humanitas University, Pieve Emanuele, Milan, Italy; 7 Colorectal Surgery, IRCCS Humanitas Research Hospital, Rozzano, Milan, Italy

## Abstract

**Importance:**

Delays in screening programs and the reluctance of patients to seek medical attention because of the outbreak of SARS-CoV-2 could be associated with the risk of more advanced colorectal cancers at diagnosis.

**Objective:**

To evaluate whether the SARS-CoV-2 pandemic was associated with more advanced oncologic stage and change in clinical presentation for patients with colorectal cancer.

**Design, Setting, and Participants:**

This retrospective, multicenter cohort study included all 17 938 adult patients who underwent surgery for colorectal cancer from March 1, 2020, to December 31, 2021 (pandemic period), and from January 1, 2018, to February 29, 2020 (prepandemic period), in 81 participating centers in Italy, including tertiary centers and community hospitals. Follow-up was 30 days from surgery.

**Exposures:**

Any type of surgical procedure for colorectal cancer, including explorative surgery, palliative procedures, and atypical or segmental resections.

**Main Outcomes and Measures:**

The primary outcome was advanced stage of colorectal cancer at diagnosis. Secondary outcomes were distant metastasis, T4 stage, aggressive biology (defined as cancer with at least 1 of the following characteristics: signet ring cells, mucinous tumor, budding, lymphovascular invasion, perineural invasion, and lymphangitis), stenotic lesion, emergency surgery, and palliative surgery. The independent association between the pandemic period and the outcomes was assessed using multivariate random-effects logistic regression, with hospital as the cluster variable.

**Results:**

A total of 17 938 patients (10 007 men [55.8%]; mean [SD] age, 70.6 [12.2] years) underwent surgery for colorectal cancer: 7796 (43.5%) during the pandemic period and 10 142 (56.5%) during the prepandemic period. Logistic regression indicated that the pandemic period was significantly associated with an increased rate of advanced-stage colorectal cancer (odds ratio [OR], 1.07; 95% CI, 1.01-1.13; *P* = .03), aggressive biology (OR, 1.32; 95% CI, 1.15-1.53; *P* < .001), and stenotic lesions (OR, 1.15; 95% CI, 1.01-1.31; *P* = .03).

**Conclusions and Relevance:**

This cohort study suggests a significant association between the SARS-CoV-2 pandemic and the risk of a more advanced oncologic stage at diagnosis among patients undergoing surgery for colorectal cancer and might indicate a potential reduction of survival for these patients.

## Key Points

**Question:**

Was the COVID-19 pandemic associated with more advanced oncologic stage at presentation for colorectal cancer?

**Findings:**

This cohort study including 17 938 patients treated for colorectal cancer at 81 Italian centers between 2018 and 2021 showed that patients who underwent surgery for colorectal cancer during the pandemic period had higher odds of diagnoses of late-stage cancer, distant metastasis, and stenotic lesions.

**Meaning:**

This study suggests that the COVID-19 pandemic was associated with diagnosis of colorectal cancer at a more advanced stage, which could potentially translate to a reduction in survival.

## Teaser

This cohort study evaluates whether the COVID-19 pandemic was associated with more advanced oncologic stage and change in clinical presentation for Italian patients with colorectal cancer.

## Introduction

The spread of SARS-CoV-2 infection and COVID-19 has resulted in an unprecedented effect on the health care system worldwide.^1^ The need to redistribute health care resources toward the treatment of the millions of patients requiring hospitalization has resulted in an unavoidable reduction in the possibility of diagnosing and treating oncologic patients.^2^ Moreover, the decreased number of cancer screening programs observed in many countries has contributed to the increased risk of a late diagnosis of colorectal cancer.^3,4,5,6^ In Italy, a decrease of more than 30% in colorectal cancer screening was observed between March 2020 and May 2021.^7^ In the US, a decrease in colonoscopies during 2020 was observed, especially among the population with a lower socioeconomic status.^8^

Although a few modeling studies predicted an increased risk of mortality because of the backlog of cancer screening and referrals,^2,9,10,11^ the association of the diagnostic delay with mortality risk has yet to be confirmed in a large population of patients, to our knowledge.^12^ The aim of this study was to address this issue by means of a multicenter national study including all patients undergoing surgery for colorectal cancer between January 1, 2018, and December 31, 2021.

## Methods

### Study Design and Participants

The COVID–Colorectal Cancer (CRC) study was a retrospective cohort study that included all consecutive patients undergoing colorectal cancer surgery between January 1, 2018, and December 31, 2021, in all 81 Italian centers participating in the study (eTable 1 in Supplement 1). No minimum number of cases was required for the centers to be eligible for registration. The study was approved by the ethical committees of the participating centers and was registered on ClinicalTrials.gov (NCT04712292). According to Italian regulations, written consent was obtained from all available patients. The present study was carried out according to the Strengthening the Reporting of Observational Studies in Epidemiology (STROBE) reporting guideline.

Inclusion criteria were age 18 years or older; colorectal surgery for cancer (including surgical radicalization after the endoscopic removal of cancerous polyps); elective or urgent surgery; palliative or curative intent; any type of surgical procedure (including explorative surgery, palliative procedures, and atypical or segmental resections) for cancer located in the colon, rectum, or anus; and a minimum follow-up of 30 days after surgery. Exclusion criteria were recurrent colorectal cancer after previous surgery or cancer originating from other organs, malignant lesions of a nature other than adenocarcinoma and squamous cell carcinoma (ie, neuroendocrine tumors, gastrointestinal stromal tumors, melanomas, lymphomas), and benign lesions.

The variables collected were identified using institutional databases, patient medical records, and operating lists, depending on the different practice in each center. The data were inserted into a REDCap (Research Electronic Data Capture; Vanderbilt University) database by a team of clinicians who were identified by the principal investigator in each participating center.^13^

The data set registered the presence of comorbidities, the details of the preoperative diagnosis, the use of preoperative chemoradiotherapy, the details of the surgery (type of operation and intraoperative complications), the presence of complications or mortality at 30 days after surgery (according to the Clavien-Dindo classification),^14^ and the histologic features.

The primary oncologic outcome was advanced cancer stage at diagnosis, defined as any of the following: (1) stage IV (M+, any T, any M), stage III (M0, N+, any T), stage IIb (T4a, N0, M0), and stage IIc (T4b, N0, M0), according to the American Joint Committee on Cancer (AJCC)^15^; (2) a case requiring palliative surgery for unresectable cancer, with or without a final histologic diagnosis or stage; or (3) a case requiring neoadjuvant chemotherapy owing to preoperative evidence of advanced clinical stage, regardless of the final histologic stage.

Secondary outcomes were (1) presence of distant metastases, diagnosed either preoperatively or postoperatively; (2) pT4 stage, regardless of the N or M stage; (3) aggressiveness of the cancer biology, defined as cancer with at least 1 of the following characteristics: signet ring cells, mucinous tumor, budding, lymphovascular invasion, perineural invasion, and lymphangitis; (4) presence of a stenotic lesion, defined as a tumor causing a narrowing of the tumor site and dilatation of the proximal bowel, correlated with symptoms of obstruction (5) urgent surgery, defined as the need to proceed to surgery within 48 hours from diagnosis or admission to the hospital; (6) palliative surgery, which included all the procedures that did not have the aim of radically removing the tumor, regardless of the timing of the decision. Surgery performed in a stage IV case was not necessarily interpreted as a palliative procedure as long as it was performed with the aim of oncologically removing the primary cancer.

The SARS-CoV-2 pandemic period was considered to be between March 1, 2020, and December 31, 2021, while the prepandemic period was between January 1, 2018, and February 29, 2020. The study period was chosen according to the increased number of patients with SARS-CoV-2 infection during the first wave of the COVID-19 pandemic, which led to a national lockdown (March 10, 2020).^16^

### Data Validation

A validator was identified in each participating center. The validator was not involved in the data collection and was responsible for checking 20% of the cases at the end of the data inclusion period (February 20, 2022). The validated cases had to be equally distributed over the 4 years of the study period. The study was carried out according to the Gantt chart shown in the eFigure in Supplement 1.

### Statistical Analysis

The continuous variables were expressed as mean (SD), while the categorical variables were presented as number and percentage. The potential association between each recorded variable and the primary outcome (advanced stage) and the 6 secondary outcomes (distant metastasis, T4 stage, aggressive biology, stenotic lesion, emergency surgery, and palliative surgery) were evaluated using the *t* test for the continuous variables and the χ^2^ test for the categorical values.

The potential independent association between the pandemic period and each of the outcomes was also assessed using multivariate random-effects logistic regression, with hospital as the cluster variable, estimating the odds ratio (OR) and 95% CI. Each model was adjusted a priori for sex, age, and location of the cancer in the rectum (vs right and left colon). All *P* values were from 2-sided tests, and results were deemed statistically significant at *P* < .05. Analyses were performed using Stata, version 15.1 (StataCorp LLC).

## Results

A total of 17 938 patients (10 007 men [55.8%]; mean [SD] age, 70.6 [12.2] years) were included in the study (Table 1), after the exclusion of 346 patients (241 with benign lesions, 35 with recurrences of cancer, and 70 with a final histologic finding of other malignant neoplasm) (Figure). The patients were treated in 81 centers (reporting to 66 different hospitals in 45 different cities). A total of 7796 patients (43.5%) underwent surgery during the SARS-CoV-2 pandemic and 10 142 (56.5%) underwent surgery before the pandemic. Table 1 shows the comparison between the 2 periods in terms of clinical characteristics and histologic details. Compared with the prepandemic period, the SARS-CoV-2 pandemic surgical period was significantly associated with a higher rate of elderly patients (aged ≥80 years: 2610 of 10 142 [25.7%] during the prepandemic period vs 2156 of 7796 [27.7%] during the pandemic period; *P* = .004), a lower rate of men (5724 of 10 142 [56.4%] during the prepandemic period vs 4283 of 7796 [54.9%] during the pandemic period; *P* = .045), a lower rate of asymptomatic patients (1941 of 10 142 [19.1%] during the prepandemic period vs 1212 of 7796 [15.6%] during the pandemic period; *P* < .001), a higher proportion of synchronous cancers (273 of 8903 [3.1%] during the prepandemic period vs 272 of 6807 [4.0%] during the pandemic period; *P* = .002), and a higher proportion of synchronous adenomas (1825 of 8869 [20.6%] during the prepandemic period vs 1547 of 6815 [22.7%] during the pandemic period; *P* = .001). In terms of AJCC tumor stages, the pandemic period was significantly associated with a lower rate of stage 1 cancer (1615 of 7796 [20.7%] during the pandemic period vs 2361 of 10 142 [23.3%] during the prepandemic period; *P* < .001) and a higher rate of stage 4 cancer (1172 of 7796 [15.0%] during the pandemic period vs 1411 of 10 142 [13.9%] during the prepandemic period; *P* = .03) compared with the prepandemic period. In addition, a trend in the association between increasing age, body mass index, and AJCC stage emerged.

**Table 1.** Characteristics of the Sample, Overall and by Period of Surgery

Variable	Overall sample (N = 17 938)	Prepandemic period (January 2018 to February 2020) (n = 10 142)	Pandemic period (March 2020 to December 2021) (n = 7796)	Difference between prepandemic and pandemic periods (95% CI)	*P* value^a^
Age, mean (SD), y	70.6 (12.2)	70.5 (12.0)	70.7 (14.0)	−0.2 (−0.5 to 0.2)	.40
Age class, No. (%)					.048^b^
<60 y	3437 (19.2)	1950 (19.2)	1487 (19.1)	0.1 (−1.0 to 1.3)	.80
60-69 y	3969 (22.1)	2286 (22.5)	1683 (21.6)	0.9 (0.3 to 2.2)	.13
70-79 y	5766 (32.1)	3296 (32.5)	2470 (31.7)	0.8 (−0.6 to 2.2)	.25
≥80 y	4766 (26.6)	2610 (25.7)	2156 (27.7)	−1.9 (−3.2 to −0.6)	.004
Sex, No. (%)					
Men	10 007 (55.8)	5724 (56.4)	4283 (54.9)	−1.5 (−3.0 to 0.0)	.045
Women	7931 (44.2)	4418 (43.6)	3513 (45.1)		
Asymptomatic disease, No. (%)	3153 (17.6)	1941 (19.1)	1212 (15.6)	3.6 (2.5 to 4.7)	<.001
Positive fecal occult blood test screening result, No./total No. (%)	4529/17 174 (26.4)	2583/9694 (26.6)	1946/7480 (26.0)	0.6 (−0.7 to 2.0)	.35
Location, No. (%)					
Right or transverse colon	7750 (43.2)	4387 (43.3)	3363 (43.1)	0.1 (−1.3 to 1.6)	.87
Left colon	5253 (29.3)	2932 (28.9)	2321 (29.8)	−0.9 (−2.2 to 0.5)	.21
Rectum	4935 (27.5)	2823 (27.8)	2112 (27.1)	0.7 (−0.6 to 2.1)	.27
Tumor histologic type					
Adenocarcinoma	17 626 (98.3)	9992 (98.5)	7634 (97.9)	0.6 (0.2 to 1.0)	.002
Squamous cell carcinoma	145 (0.8)	76 (0.8)	69 (0.9)	−0.1 (−0.4 to 0.1)	.31
No histology (palliative surgery)	167 (0.9)	74 (0.7)	93 (1.2)	−0.5 (−0.7 to −0.2)	.001
AJCC tumor stage, No. (%)					.005^b^
0	523 (2.9)	302 (3.0)	221 (2.8)	0.1 (−0.4 to 0.6)	.57
1	3976 (22.2)	2361 (23.3)	1615 (20.7)	2.6 (1.3 to 3.8)	<.001
2a	4598 (25.6)	2550 (25.1)	2048 (26.3)	−1.1 (−2.4 to 0.2)	.09
2b-c	764 (4.3)	408 (4.0)	356 (4.6)	−0.5 (−1.1 to 0.1)	.07
3a	568 (3.2)	342 (3.4)	226 (2.9)	0.5 (0.0 to 1.0)	.07
3b	3351 (18.7)	1908 (18.8)	1443 (18.5)	0.3 (−0.8 to 1.5)	.61
3c	1083 (6.0)	592 (5.8)	491 (6.3)	−0.5 (−1.2 to 0.2)	.20
4	2583 (14.4)	1411 (13.9)	1172 (15.0)	−1.1 (−2.2 to −0.1)	.03
No stage^c^	492 (2.7)	268 (2.6)	224 (2.9)	−0.2 (−0.7 to 0.3)	.35
Synchronous cancers, No./total No. (%)	545/15 710 (3.5)	273/8903 (3.1)	272/6807 (4.0)	−0.9 (−1.5 to −0.4)	.002
Synchronous adenomas, No./total No. (%)	3372/15 684 (21.5)	1825/8869 (20.6)	1547/6815 (22.7)	−2.1 (−3.4 to −0.8)	.001
BMI, mean (SD)	25.6 (4.8)	25.6 (4.8)	25.5 (4.9)	0.1 (0.0 to 0.3)	.09
BMI category, No./total No. (%)					.02^b^
<18	218/14 295 (1.5)	120/8067 (1.5)	98/6228 (1.6)	−0.1 (−0.5 to 0.3)	.68
18-24	5976/14 295 (41.8)	3312/8067 (41.1)	2664/6228 (42.8)	−1.7 (−3.3 to −0.1)	.04
25-29	5909/14 295 (41.3)	3362/8067 (41.7)	2547/6228 (40.9)	0.8 (−0.1 to 2.4)	.35
30-34	1680/14 295 (11.8)	976/8067 (12.1)	704/6228 (11.3)	0.8 (−0.3 to 1.9)	.14
≥35	512/14 295 (3.6)	297/8067 (3.7)	215/6228 (3.5)	0.2 (−0.4 to 0.8)	.46
Primary outcome, No. (%)					
Advanced stage	8841 (49.3)	4929 (48.6)	3912 (50.2)	−1.5 (−3.1 to −0.1)	.04
Secondary outcomes, No. (%)					
Distant metastasis	2583 (14.4)	1411 (13.9)	1172 (15.0)	−1.1 (−2.2 to −0.1)	.03
T4	1450 (8.1)	758 (7.5)	692 (8.9)	−1.4 (−2.2 to −0.6)	.001
Stenotic lesion	2611 (14.6)	1396 (13.8)	1215 (15.6)	−1.8 (−2.9 to −0.8)	.001
Urgent surgery	2025 (11.3)	1076 (10.6)	949 (12.2)	−1.6 (−2.5 to −0.6)	.001
Palliative surgery	1379 (7.7)	735 (7.3)	644 (8.3)	−1.0 (−1.8 to −0.2)	.01
Aggressive biology, No./total No. (%)	12 207/17 446 (70.0)	6656/9874 (67.4)	5551/7572 (73.3)	−5.9 (−7.3 to −4.5)	<.001

Abbreviations: AJCC, American Joint Committee on Cancer; BMI, body mass index (calculated as weight in kilograms divided by height in meters squared).

^a^ *t* Test and χ^2^ test for continuous and categorical variables, respectively.

^b^ *P* value for trend.

^c^ No stage included cancers that were not removed by palliative surgery or that had a pathologic complete response after neoadjuvant therapy.

**Figure.**
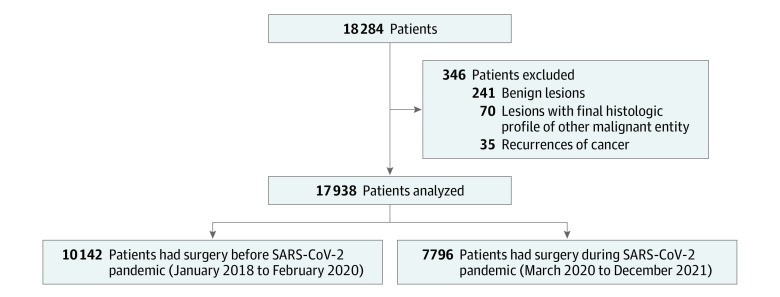
Flow Chart of the Study

The comparison of the primary and the secondary outcomes according to the period of surgery is also reported in Table 1. A significant increase in cases at an advanced stage (3912 of 7796 [50.2%] during the pandemic period vs 4929 of 10 142 [48.6%] during the prepandemic period; *P* = .04), of metastatic disease (1172 of 7796 [15.0%] during the pandemic period vs 1411 of 10 142 [13.9%] during the prepandemic period; *P* = .03), T4 stage cancers (692 of 7796 [8.9%] during the pandemic period vs 758 of 10 142 [7.5%] during the prepandemic period; *P* = .001), of tumors with an aggressive biology (5551 of 7572 [73.3%] during the pandemic period vs 6656 of 9874 [67.4%] during the prepandemic period; *P* < .001), of stenotic lesions (1215 of 7796 [15.6%] during the pandemic period vs 1396 of 10 142 [13.8%] during the prepandemic period; *P* = .001), of urgent surgery (949 of 7796 [12.2%] during the pandemic period vs 1076 of 10 142 [10.6%] during the prepandemic period; *P* = .001), and palliative surgery (644 of 7796 [8.3%] during the pandemic period vs 735 of 10 142 [7.3%] during the prepandemic period; *P* = .01) was observed during the SARS-CoV-2 pandemic period compared with the prepandemic period.

Table 2 reports the comparison of the clinical characteristics and the histologic details according to the primary outcome of advanced disease stage. Tumors at an advanced stage were significantly associated with patients younger than 60 years (1859 of 8841 [21.0%] vs 1578 of 9097 [17.4%]; *P* < .001) and with left colon (2676 of 8841 [30.3%] vs 2577 of 9097 [28.3%]; *P* = .004) and rectum (2493 of 8841 [28.2%] vs 2442 of 9097 [26.8%]; *P* = .04) locations. Similar analyses according to the secondary outcomes are reported in eTables 2 to 7 in Supplement 1.

**Table 2.** Comparison of the Clinical Characteristics and the Histologic Variables According to the Primary Outcome of Advanced Stage

Variable	Patients, No./total No. (%)		*P* value^a^
	Not advanced stage	Advanced stage	
Age, mean (SD), y	71.1 (11.8)	70.0 (12.6)	<.001
Age class, y			
<60	1578/9097 (17.4)	1859/8841 (21.0)	<.001
60-69	2036/9097 (22.4)	1933/8841 (21.9)	.40
70-79	2999/9097 (33.0)	2767/8841 (31.3)	.02
≥80	2484/9097 (27.3)	2282/8841 (25.8)	.02
Male sex	4021/9097 (44.2)	3910/8841 (44.2)	.97
Asymptomatic disease	2041/9097 (22.4)	1112 (12.6)	<.001
Positive fecal occult blood test screening result	2634/8721 (30.2)	1895/8453 (22.4)	<.001
Location			
Right or transverse colon	4078/9097 (44.8)	3672/8841 (41.5)	<.001
Left colon	2577/9097 (28.3)	2676/8841 (30.3)	.004
Rectum	2442/9097 (26.8)	2493/8841 (28.2)	.04
Tumor histologic type			
Adenocarcinoma	9029/9097 (99.3)	8597/8841 (97.2)	.002
Squamous cell carcinoma	68/9097 (0.7)	77/8841 (0.9)	.27
No histology (palliative surgery)	0	167/8841 (1.9)	<.001
SARS-CoV-2 pandemic period	3884/9097 (42.7)	3912/8841 (44.3)	.04
Synchronous cancers	270/8312 (3.3)	275/7398 (3.7)	.11
Synchronous adenomas	2036/8571 (23.8)	1336/7376 (18.1)	<.001
BMI, mean (SD)	25.8 (4.8)	25.4 (4.9)	<.001
BMI category			
<18	83/7331 (1.1)	135/6964 (1.9)	<.001
18-24	2971/7331 (40.5)	3005/6964 (43.2)	.001
25-29	3081/7331 (42.0)	2828/6964 (40.6)	.09
30-34	912/7331 (12.4)	768/6964 (11.0)	.009
≥35	284/7331 (3.9)	228/6964 (3.3)	.05

Abbreviation: BMI, body mass index (calculated as weight in kilograms divided by height in meters squared).

^a^ *t* Test and χ^2^ test for continuous and categorical variables, respectively.

The results of the random-effects logistic regression analyses are shown in Table 3. Surgery performed during the SARS-CoV-2 pandemic period was significantly associated with advanced disease stage (OR, 1.07; 95% CI, 1.01-1.13; *P* = .03), aggressive biology (OR, 1.32; 95% CI, 1.15-1.53; *P* < .001), and stenotic lesions (OR, 1.15; 95% CI, 1.01-1.31; *P* = .03), while no significant association was found between the pandemic period and distant metastases (OR, 1.10; 95% CI, 1.00-1.21; *P* = .05), T4 stage (OR, 1.20; 95% CI, 0.99-1.45; *P* = .06), urgent surgery (OR, 1.15; 95% CI, 0.96-1.39; *P* = .12), and palliative surgery (OR, 1.15; 95% CI, 0.96-1.37; *P* = .12).

**Table 3.** Random-Effects Multivariate Logistic Regression Models, With Hospital as the Cluster Variable, Assessing the Association Between Independent Variables and the Primary Outcome (Advanced Stage) and 6 Secondary Outcomes^a^

Variable	Advanced stage		Distant metastasis		T4 stage		Aggressive biology		Stenotic lesion		Urgent surgery			Palliative surgery	
	AOR (95% CI) (n = 17 938)	*P* value^b^	AOR (95% CI) (n = 17 938)	*P* value^b^	AOR (95% CI) (n = 17 872)	*P* value^b^	AOR (95% CI) (n = 17 446)	*P* value^b^	AOR (95% CI) (n = 17 938)	*P* value^b^	AOR (95% CI) (n = 17 938)		*P* value^b^	AOR (95% CI) (n = 17 938)	*P* value^b^
SARS-CoV-2 pandemic period	1.07 (1.01-1.13)	.03	1.10 (1.00-1.21)	.05	1.20 (0.99-1.45)	.06	1.32 (1.15-1.53)	<.001	1.15 (1.01-1.31)	.03	1.15 (0.96-1.39)	.12		1.15 (0.96-1.37)	.12
Male sex	1.00 (0.93-1.06)	.92	1.02 (0.94-1.12)	.57	0.78 (0.71-0.85)	<.001	0.97 (0.89-1.05)	.47	1.04 (0.96-1.12)	.38	0.87 (0.78-0.96)	.006		1.01 (0.89-1.14)	.88
Age <60 y (vs ≥60 y)	1.20 (1.08-1.33)	.001	0.96 (0.85-1.08)	.47	1.11 (0.88-1.39)	.38	1.26 (1.10-1.43)	<.001	1.30 (1.12-1.51)	.001	2.03 (1.66-2.49)	<.001		1.60 (1.26-2.04)	<.001
Rectum (vs right, transverse, and left colon)	1.05 (0.97-1.13)	.27	0.99 (0.89-1.09)	.78	1.17 (1.00-1.37)	.047	0.59 (0.53-0.66)	<.001	0.60 (0.50-0.72)	<.001	0.47 (0.39-0.57)	<.001		0.96 (0.80-1.14)	.62

Abbreviation: AOR, adjusted odds ratio.

^a^ Likelihood ratio test for goodness-of-fit *P* < .001 in all models.

^b^ Wald test.

## Discussion

Since January 2020, the COVID-19 pandemic has caused more than 529 million confirmed cases and more than 6 million deaths.^1^ In Italy, 17 896 065 cases and 167 780 deaths have been reported,^17^ while 85 263 864 cases and 1 003 740 deaths have been confirmed in the US.^18^ During the past 2 years, health care systems have experienced unprecedented backlogs of oncologic procedures worldwide, owing to a reduction in referral pathways and screening programs that became necessary in view of the rapid increase in the number of patients with COVID-19 who required acute treatment in the hospital.^19^ In addition, patients have been reluctant to seek medical care for the same symptoms that, prior to the pandemic, would have prompted an appointment with their primary care physician or at the hospital.^20^

Based on these reports, modeling studies showed predictions of increased mortality owing to the delayed diagnosis of colorectal cancer.^8,9,10,11^ Nevertheless, the association between the COVID-19 pandemic period and the deterioration of the oncologic outcomes of patients with colorectal cancer has yet to be confirmed, to our knowledge. Some results have been provided by single-center studies, although they were limited by the small volume of cases or the short periods of observation. Eklöv et al^21^ reported a significantly higher rate of stage T4 cancer among 550 patients with colorectal cancer in 2020 compared with 590 patients treated in 2019. A previous study from the COVID-CRC Collaborative group analyzed 3236 patients and found an increase of clinical T4 stage and of multiple liver metastases.^12^ Although those characteristics were hypothesized to be potential factors associated with worse oncologic outcomes,^22,23,24^ the study failed to find any association between the pandemic period and more advanced tumor stages.

The present study analyzed 17 938 patients who were treated over a consecutive period of 4 years and detected a significant association between the SARS-CoV-2 pandemic period of surgery and advanced stage of cancer at diagnosis (OR, 1.07; 95% CI, 1.01-1.13; *P* = .03), after adjustment for confounding factors (age, sex, and location of the cancer). In particular, analysis of the secondary outcomes showed that patients who underwent surgery during the SARS-CoV-2 pandemic period had a greater (although not statistically significant) risk of receiving a diagnosis of distant metastases (OR, 1.10; 95% CI, 1.00-1.21; *P* = .05), which represents a worrisome finding, because metastatic disease is strongly associated with cancer-related mortality.^15^ The number of patients who received a diagnosis of metastatic disease during the pandemic period increased by 8.6% compared with the expected rate based on the previous period. Taking into account the prevalence of colorectal cancer in Western countries,^25^ the present study might indicate a large increase in the rates of metastatic cancer at diagnosis, if the present results reflect a similar trend among the overall population. Evidence regarding the rapid timeline of the development of distant metastases of colorectal cancer supports these findings.^26,27,28^

### Limitations and Strengths

The present study has some limitations. First, its retrospective nature did not allow retrieving the exact timing between the onset of symptoms and the diagnosis, or between diagnosis and surgical treatment. Second, the study collected the data from only the centers that participated voluntarily; therefore, the study population may not represent the general population of patients undergoing surgery for colorectal cancer in Italy. Third, there is a proportion of patients with colorectal cancer who were not included in the present study; for instance, those who received a diagnosis of nonoperable cancer who received only medical palliative treatment.

This study also has some strengths. It presented a large series of patients undergoing oncologic colorectal surgery during the past 4 years, including an observation of 22 months of the COVID-19 pandemic period, from a clinical scenario because it included tertiary centers and community hospitals, as well as elective and urgent surgery, regardless of the case volume of the centers. The wide variation of the outcomes among the centers was adjusted using multivariate random-effects logistic regression, which had hospitals as the cluster variable. The extended period (4 years) and the large number of patients were likely to reduce the bias associated with the random variations of the oncologic outcomes over the years.

## Conclusions

To our knowledge, this is the largest national multicenter study to date reporting a significant association between the onset of the COVID-19 pandemic and the worsening of the oncologic stage at diagnosis among a large series of patients undergoing surgery for colorectal cancer. This evidence, which might reflect a similar situation in other countries that also experienced a backlog of the screening and diagnostic procedures, could result in the first decrease in survival rates to be observed in the past 2 decades among patients with colorectal cancer.^29^ Once again, the evidence of the present study points out the importance of a large-scale response that might mitigate the detrimental association between the COVID-19 pandemic and the worsened oncologic outcomes of patients with colorectal cancer.

## Notes

Supplement 1. **eFigure.** Gantt Chart Displaying the Schedule of the Study Over the Period **eTable 1.** Outcomes for Each Hospital (Cluster) Included in the Study **eTable 2.** Comparison of the Clinical Characteristics and the Histological Variables According to the Secondary Outcome Distant Metastasis **eTable 3.** Comparison of the Clinical Characteristics and the Histological Variables According to the Secondary Outcome T4 Stage **eTable 4.** Comparison of the Clinical Characteristics and the Histological Variables According to the Secondary Outcome Aggressive Biology **eTable 5.** Comparison of the Clinical Characteristics and the Histological Variables According to the Secondary Outcome Stenotic Tumor **eTable 6.** Comparison of the Clinical Characteristics and the Histological Variables According to the Secondary Outcome Urgent Surgery **eTable 7.** Comparison of the Clinical Characteristics and the Histological Variables According to the Secondary Outcome Palliative Surgery

Supplement 2. **Nonauthor Collaborators.** COVID–Colorectal Cancer (CRC) Study Group

Supplement 3.
Data Sharing Statement
