# Supplementary material for: Colorectal Cancer Stage at Diagnosis Before vs During the COVID-19 Pandemic in Italy
Source: JAMA Netw Open. 2022 Nov 21;5(11):e2243119. doi: 10.1001/jamanetworkopen.2022.43119 (PMC9679872; doi:10.1001/jamanetworkopen.2022.43119)
Supplement: Supplement 1. — eFigure. Gantt Chart Displaying the Schedule of the Study Over the Period eTable 1. Outcomes for Each Hospital (Cluster) Included in the Study eTable 2. Comparison of the Clinical Characteristics and the Histological Variables According to the Secondary Outcome Distant Metastasis eTable 3. Comparison of the Clinical Characteristics and the Histological Variables According to the Secondary Outcome T4 Stage eTable 4. Comparison of the Clinical Characteristics and the Histological Variables According to the Secondary Outcome Aggressive Biology eTable 5. Comparison of the Clinical Characteristics and the Histological Variables According to the Secondary Outcome Stenotic Tumor eTable 6. Comparison of the Clinical Characteristics and the Histological Variables According to the Secondary Outcome Urgent Surgery eTable 7. Comparison of the Clinical Characteristics and the Histological Variables According to the Secondary Outcome Palliative Surgery [file jamanetwopen-e2243119-s001.pdf]

Supplementary Online Content

Rottoli M, Gori A, Pellino G, et al; COVID–Colorectal Cancer (CRC) Study Group. Colorectal cancer stage at diagnosis before vs during the COVID-19 pandemic in Italy. *JAMA Netw Open.* 2022;5(11):e2243119. doi:10.1001/jamanetworkopen.2022.43119

eFigure. Gantt Chart Displaying the Schedule of the Study Over the Period
eTable 1. Outcomes for Each Hospital (Cluster) Included in the Study
eTable 2. Comparison of the Clinical Characteristics and the Histological Variables According to the Secondary Outcome Distant Metastasis
eTable 3. Comparison of the Clinical Characteristics and the Histological Variables According to the Secondary Outcome T4 Stage
eTable 4. Comparison of the Clinical Characteristics and the Histological Variables According to the Secondary Outcome Aggressive Biology
eTable 5. Comparison of the Clinical Characteristics and the Histological Variables According to the Secondary Outcome Stenotic Tumor
eTable 6. Comparison of the Clinical Characteristics and the Histological Variables According to the Secondary Outcome Urgent Surgery
eTable 7. Comparison of the Clinical Characteristics and the Histological Variables According to the Secondary Outcome Palliative Surgery

This supplementary material has been provided by the authors to give readers additional information about their work.

© 2022 Rottoli M et al. *JAMA Network Open.*

eFigure. Gantt Chart Displaying the Schedule of the Study Over the Period

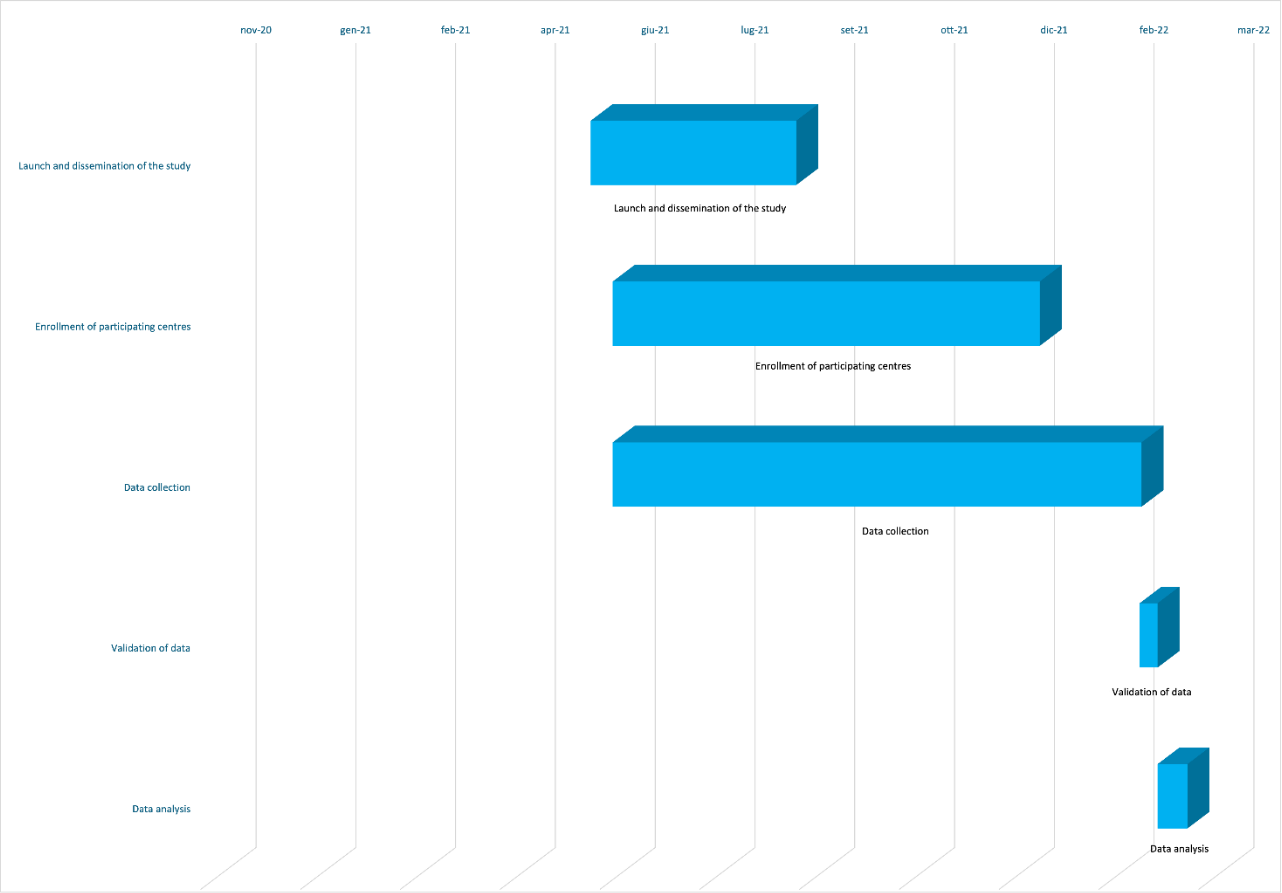

© 2022 Rottoli M et al. *JAMA Network Open.*

eTable 1. Outcomes for Each Hospital (Cluster) Included in the Study

Hospital ID	Total N of cases	Advanced stage, N (%)	T4, N (%)	Metastasis, N (%)	Aggressive biology, N (%)	Stenosing lesion, N (%)	Critical conditions, N (%)	Palliative surgery, N (%)
Hospital ID 1	186	88 (47.3)	3 (1.7)	32 (17.2)	82 (44.3)	0 (0.0)	1 (0.5)	3 (1.6)
Hospital ID 2	281	133 (47.3)	20 (7.1)	28 (10.0)	230 (82.4)	1 (0.4)	87 (31.0)	23 (8.2)
Hospital ID 3	185	98 (53.0)	19 (10.3)	38 (20.5)	102 (55.1)	18 (9.7)	15 (8.1)	23 (12.4)
Hospital ID 4	256	132 (51.6)	15 (5.9)	23 (9.0)	175 (68.9)	42 (16.4)	32 (12.5)	11 (4.3)
Hospital ID 5	683	338 (49.5)	66 (9.7)	107 (15.7)	460 (68.8)	141 (20.6)	125 (18.3)	46 (6.7)
Hospital ID 6	488	219 (44.8)	38 (7.8)	71 (14.6)	366 (78.0)	82 (16.8)	8 (1.6)	32 (6.6)
Hospital ID 7	135	84 (62.2)	16 (12.2)	24 (17.8)	124 (94.7)	29 (21.5)	34 (25.2)	19 (14.1)
Hospital ID 8	135	67 (49.6)	5 (3.8)	23 (17.0)	115 (97.1)	31 (23.0)	28 (20.7)	9 (6.7)
Hospital ID 9	634	348 (54.9)	43 (6.8)	92 (14.5)	484 (80.5)	97 (15.3)	78 (12.3)	25 (3.9)
Hospital ID 10	556	271 (48.7)	78 (14.0)	83 (14.9)	417 (78.2)	43 (7.7)	33 (5.9)	61 (11.0)
Hospital ID 11	222	114 (51.4)	18 (8.1)	39 (17.6)	168 (76.7)	47 (21.2)	30 (13.5)	18 (8.1)
Hospital ID 12	587	273 (46.5)	51 (8.7)	85 (14.5)	392 (71.7)	119 (20.3)	6 (1.0)	31 (5.3)
Hospital ID 13	95	63 (33.7)	14 (14.9)	29 (30.5)	74 (80.4)	7 (7.4)	51 (53.7)	20 (21.1)
Hospital ID 14	195	94 (66.3)	17 (5.9)	28 (14.4)	83 (42.6)	38 (19.5)	12 (6.2)	20 (10.3)
Hospital ID 15	80	37 (46.3)	11 (13.8)	11 (13.8)	56 (71.8)	6 (7.5)	10 (12.5)	7 (8.8)
Hospital ID 16	141	70 (49.7)	17 (12.1)	9 (6.4)	104 (73.8)	13 (9.2)	20 (14.2)	13 (9.2)
Hospital ID 17	120	74 (61.7)	40 (33.3)	33 (27.5)	94 (79.7)	23 (19.2)	42 (35.0)	32 (26.7)
Hospital ID 18	445	224 (50.3)	51 (11.5)	57 (12.8)	341 (77.5)	124 (27.9)	33 (7.4)	18 (4.0)
Hospital ID 19	94	51 (54.3)	12 (12.8)	17 (18.1)	45 (48.9)	13 (13.8)	11 (11.7)	10 (10.6)
Hospital ID 20	167	73 (43.7)	18 (10.8)	25 (15.0)	55 (34.0)	41 (24.6)	2 (1.2)	13 (7.8)
Hospital ID 21	137	71 (51.8)	10 (7.3)	18 (13.1)	115 (87.1)	27 (19.7)	8 (5.8)	12 (8.8)
Hospital ID 22	324	166 (51.2)	28 (8.7)	44 (13.6)	169 (54.9)	63 (19.4)	54 (16.7)	39 (12.0)
Hospital ID 23	114	58 (50.9)	8 (7.1)	22 (19.3)	96 (85.7)	24 (21.1)	2 (1.8)	6 (5.3)
Hospital ID 24	130	56 (43.1)	15 (11.5)	16 (12.3)	58 (44.6)	4 (3.1)	14 (10.8)	5 (3.8)
Hospital ID 25	312	160 (51.3)	19 (6.3)	32 (10.3)	178 (58.6)	46 (14.7)	98 (31.1)	39 (12.5)
Hospital ID 26	130	66 (50.8)	19 (14.6)	38 (29.2)	102 (79.1)	27 (20.8)	47 (36.2)	20 (15.4)
Hospital ID 27	448	201 (44.9)	21 (4.7)	62 (13.8)	251 (58.9)	24 (5.4)	7 (1.6)	13 (2.9)
Hospital ID 28	236	116 (49.2)	10 (4.2)	29 (12.3)	120 (54.3)	23 (9.8)	2 (0.9)	5 (2.1)
Hospital ID 29	279	137 (49.1)	16 (5.8)	45 (16.1)	203 (74.4)	54 (19.4)	18 (6.5)	20 (7.2)
Hospital ID 30	104	53 (51.0)	2 (1.9)	11 (10.6)	94 (90.4)	17 (16.4)	12 (11.5)	7 (6.7)
Hospital ID 31	255	138 (54.1)	23 (9.1)	44 (17.3)	174 (70.2)	40 (15.7)	31 (12.2)	25 (9.8)
Hospital ID 32	530	260 (49.1)	40 (7.6)	67 (12.6)	371 (72.2)	61 (11.5)	17 (3.2)	16 (3.0)
Hospital ID 33	151	72 (47.7)	4 (2.7)	24 (15.9)	101 (66.9)	19 (12.6)	8 (5.3)	2 (1.3)
Hospital ID 34	272	121 (44.5)	15 (5.5)	24 (8.8)	218 (81.0)	19 (7.0)	20 (7.4)	14 (5.2)
Hospital ID 35	285	114 (40.0)	17 (6.0)	29 (10.2)	140 (49.1)	53 (18.6)	8 (2.8)	11 (3.9)
Hospital ID 36	130	57 (43.9)	10 (7.7)	10 (7.7)	79 (69.3)	2 (1.5)	12 (9.2)	0 (0.0)
Hospital ID 37	203	95 (46.8)	15 (7.4)	15 (7.4)	152 (76.0)	29 (14.3)	12 (5.9)	8 (3.9)
Hospital ID 38	282	148 (52.5)	7 (2.5)	164 (15.3)	214 (77.0)	28 (9.9)	0 (0.0)	21 (7.5)
Hospital ID 39	852	442 (51.9)	81 (9.5)	8 (19.3)	656 (81.0)	133 (15.6)	54 (6.3)	77 (9.0)
Hospital ID 40	186	82 (44.1)	0 (0.0)	46 (4.3)	109 (58.9)	0 (0.0)	10 (5.4)	7 (3.8)
Hospital ID 41	298	173 (58.1)	32 (10.9)	19 (15.4)	240 (83.0)	43 (14.4)	49 (16.4)	30 (10.1)
Hospital ID 42	120	67 (55.8)	16 (13.3)	42 (15.8)	50 (42.7)	32 (26.7)	24 (20.0)	10 (8.3)
Hospital ID 43	378	191 (50.5)	25 (6.7)	31 (11.1)	193 (51.2)	52 (13.8)	84 (22.2)	36 (9.5)
Hospital ID 44	281	118 (42.0)	15 (5.3)	65 (11.0)	202 (71.1)	15 (5.3)	29 (10.3)	6 (2.1)
Hospital ID 45	523	250 (47.8)	36 (6.9)	51 (12.4)	403 (81.3)	78 (14.9)	10 (1.9)	44 (8.4)
Hospital ID 46	264	139 (52.7)	14 (5.3)	30 (19.3)	209 (79.2)	87 (33.0)	49 (18.6)	37 (14.0)
Hospital ID 47	237	126 (53.2)	17 (7.2)	13(12.7)	204 (86.4)	27 (11.4)	54 (22.8)	11 (4.6)
Hospital ID 48	261	114 (43.7)	18 (6.9)	13 (5.0)	163 (64.7)	26 (10.0)	18 (6.9)	11 (4.2)
Hospital ID 49	181	74 (40.9)	2 (1.1)	14 (7.7)	147 (83.5)	22 (12.2)	1 (0.6)	8 (4.4)
Hospital ID 50	106	53 (50.0)	15 (14.2)	13 (12.3)	71 (68.3)	11 (10.4)	19 (17.9)	10 (9.4)
Hospital ID 51	130	85 (65.4)	20 (15.4)	27 (20.8)	85 (65.4)	10 (7.7)	26 (20.0)	13 (10.0)
Hospital ID 52	197	76 (38.6)	6 (3.0)	10 (5.1)	72 (36.7)	11 (5.6)	12 (6.1)	5 (2.5)
Hospital ID 53	200	121 (60.5)	19 (9.7)	30 (15.0)	179 (90.4)	6 (3.0)	18 (9.0)	11 (5.5)
Hospital ID 54	380	168 (44.2)	13 (3.4)	70 (18.4)	179 (48.5)	92 (24.2)	27 (7.1)	48 (12.6)
Hospital ID 55	157	77 (49.0)	26 (17.0)	23 (14.7)	112 (71.8)	30 (19.1)	47 (29.9)	17 (10.8)
Hospital ID 56	177	82 (46.3)	10 (5.7)	17 (9.6)	87 (49.4)	13 (7.3)	30 (17.0)	19 (10.7)
Hospital ID 57	525	231 (44.0)	20 (3.8)	49 (9.3)	400 (78.3)	70 (13.3)	17 (3.2)	9 (1.7)
Hospital ID 58	455	253 (55.6)	52 (11.4)	118 (25.9)	372 (82.3)	66 (14.5)	91 (20.0)	31 (6.8)
Hospital ID 59	102	37 (36.3)	6 (5.9)	10 (9.8)	75 (75.0)	8 (7.8)	2 (2.0)	4 (3.9)
Hospital ID 60	163	83 (50.9)	17 (10.5)	35 (21.5)	126 (80.8)	8 (4.9)	51 (31.3)	20 (12.3)
Hospital ID 61	369	183 (49.6)	50 (13.6)	41 (11.1)	245 (69.4)	67 (18.2)	65 (17.6)	29 (4.9)
Hospital ID 62	315	155 (49.2)	29 (9.2)	54 (17.1)	151 (50.0)	54 (17.1)	67 (21.3)	64 (20.3)
Hospital ID 63	571	271 (47.5)	57 (10.1)	85 (14.9)	300 (54.6)	107 (18.7)	52 (9.1)	50 (8.8)
Hospital ID 64	232	121 (52.2)	15 (6.5)	52 (22.4)	167 (73.3)	46(19.8)	44 (19.0)	66 (28.5)
Hospital ID 65	120	42 (35.0)	3 (2.5)	10 (8.3)	81 (68.6)	16 (13.3)	29 (24.2)	2 (1.7)
Hospital ID 66	151	87 (57.6)	5 (3.3)	29 (19.2)	127 (87.0)	6 (4.0)	18 (11.9)	7 (4.6)

© 2022 Rottoli M et al. *JAMA Network Open.*

eTable 2. Comparison of the Clinical Characteristics and the Histological Variables According to the Secondary Outcome Distant Metastasis

**Variables**	**Overall sample (N=17 938)**	**No distant metastasis (N=15 355)**	**Distant metastasis (N=2583)**	**p***
Mean age in years (SD)	70.6 (12.2)	70.9 (12.1)	68.5 (12.8)	<0.001
Age class, N (%)				
- <60	3437 (19.2)	2816 (18.3)	621 (24.0)	<0.001
- 60-69	3969 (22.1)	3347 (21.8)	622 (24.1)	0.010
- 70-79	5766 (32.1)	4992 (32.5)	774 (30.0)	0.010
- ≥80	4766 (26.6)	4200 (27.4)	566 (21.9)	<0.001
Male sex, N (%)	10 007 (55.8)	8547 (55.7)	1460 (56.5)	0.42
Asymptomatic, N (%)	3135 (17.6)	2892 (18.8)	261 (10.1)	<0.001
Positive fecal occult blood test screening, N (%)	4529 (26.4)	4104 (28.0)	425 (17.1)	<0.001
Location, N (%)				
- Right or transverse	7750 (43.2)	6689 (43.6)	1061 (41.1)	0.02
- Left	5253 (29.3)	4460 (29.1)	793 (30.7)	0.09
- Rectum	4953 (27.5)	4206 (27.4)	729 (28.2)	0.4
Tumor histological type, N (%)				
- Adenocarcinoma	17 626 (98.3)	15 142 (98.6)	2484 (96.2)	<0.001
- Squamous cell carcinoma	145 (0.8)	126 (0.8)	19 (0.7)	0.66
- No histology for palliative surgery	167 (0.9)	87 (0.6)	80 (3.1)	<0.001
SARS-CoV-2 pandemic period, N (%)	7796 (43.5)	6624 (43.1)	1172 (45.4)	0.03
	(N=15 710)	(N=13 601)	(N=2109)	
Synchronous cancers, N (%)	545 (3.5)	455 (3.3)	90 (4.3)	0.03
	(N=15 684)	(N=13 577)	(N=2107)	
Synchronous adenomas, N (%)	3372 (21.5)	3072 (22.6)	300 (14.2)	<0.001
	(N=14 295)	(N=12,273)	(N=2022)	
BMI, mean (SD)	25.6 (4.8)	25.7 (4.7)	25.1 (4.7)	<0.001
BMI category, N (%)				
- <18	218 (1.5)	167 (1.4)	51 (2.5)	<0.001
≥18 and <25	5976 (41.8)	5033 (41.0)	943 (46.6)	<0.001
- ≥25 and <30	5909 (41.3)	5134 (41.8)	775 (38.3)	0.003
- ≥30 and <35	1680 (11.8)	1491 (12.2)	189 (9.4)	<0.001
- ≥35	512 (3.6)	448 (3.7)	64 (3.2)	0.28

* T-test and Chi-squared test for continuous and categorical variables, respectively. BMI: body mass index; SD: Standard Deviation.

© 2022 Rottoli M et al. *JAMA Network Open.*

eTable 3. Comparison of the Clinical Characteristics and the Histological Variables According to the Secondary Outcome T4 Stage

**Variables**	**Overall sample (N=17 872)**	**T0-T3 (N=16 422)**	**T4 (N=1450)**	**p***
Mean age in years (SD)	70.5 (12.2)	70.6 (12.2)	69.9 (13.0)	0.12
Age class, N (%)				
- <60	3426 (19.2)	3114 (19.0)	312 (21.5)	0.018
- 60-69	3955 (22.1)	3631 (22.1)	324 (22.3)	0.84
- 70-79	5748 (32.1)	5339 (32.5)	409 (28.2)	0.001
- ≥80	4743 (26.6)	4338 (26.4)	405 (27.9)	0.21
Male sex, N (%)	9971 (55.8)	9246 (56.3)	725 (50.0)	<0.001
Asymptomatic, N (%)	3135 (17.5)	3034 (18.5)	101 (7.0)	<0.001
Positive fecal occult blood test screening, N (%)	4513 (26.4)	4316 (27.5)	197 (14.1)	<0.001
Location, N (%)				
- Right or transverse	7715 (43.2)	7118 (43.3)	597 (41.2)	0.11
- Left	5238 (29.3)	4826 (29.4)	412 (28.4)	0.44
- Rectum	4919 (27.5)	4478 (27.3)	441 (30.4)	0.01
Tumor histological type, N (%)				
- Adenocarcinoma	17 562 (98.3)	16 204 (98.7)	1358 (93.7)	<0.001
- Squamous cell carcinoma	144 (0.8)	117 (0.7)	27 (1.9)	<0.001
- No histology for palliative surgery	166 (0.9)	101 (0.6)	65 (4.5)	<0.001
SARS-CoV-2 pandemic period, N (%)	7776 (43.5)	7084 (43.1)	(692 47.7)	0.001
	(N=15 666)	(N=14 580)	(N=1086)	
Synchronous cancers, N (%)	542 (3.4)	497 (4.1)	45 (3.5)	0.20
	(N=15 639)	(N=14 553)	(N=1086)	
Synchronous adenomas, N (%)	3358 (22.1)	3218 (12.9)	140 (21.5)	<0.001
	(N=14 259)	(N=13 101)	(N=1158)	
BMI, mean (SD)	25.6 (4.8)	25.7 (4.8)	24.4 (4.9)	<0.001
BMI category, N (%)				
- <18	217 (1.5)	176 (1.3)	41 (3.5)	<0.001
- ≥18 and <25	5964 (41.8)	5369 (41.0)	595 (51.4)	<0.001
- ≥25 and <30	5894 (41.3)	5489 (41.9)	405 (35.0)	<0.001
- ≥30 and <35	1674 (11.7)	1585 (12.1)	89 (7.7)	<0.001
- ≥35	510 (3.6)	482 (3.7)	28 (2.4)	0.027

* T-test and Chi-squared test for continuous and categorical variables, respectively. BMI: body mass index; SD: Standard Deviation.

© 2022 Rottoli M et al. *JAMA Network Open.*

eTable 4. Comparison of the Clinical Characteristics and the Histological Variables According to the Secondary Outcome Aggressive Biology

**Variables**	**Overall sample (N=17 446)**	**Not aggressive biology (N=5239)**	**Aggressive biology (N=12 207)**	**p***
Mean age in years (SD)	70.7 (12.2)	69.7 (11.9)	71.1 (12.3)	<0.001
Age class, N (%)				
- <60	3292 (18.9)	1065 (20.3)	2227 (18.2)	0.001
- 60-69	9824 (21.9)	1289 (24.6)	2535 (20.8)	<0.001
- 70-79	5642 (32.3)	1687 (32.2)	3955 (32.4)	0.80
- ≥80	4688 (26.9)	1198 (22.9)	3490 (28.6)	<0.001
Male sex, N (%)	9719 (55.7)	2985 (57.0)	6734 (55.2)	0.025
Asymptomatic, N (%)	3087 (17.7)	1223 (23.4)	1864 (15.3)	<0.001
Positive fecal occult blood test screening, N (%)	4434 (26.6)	1433 (28.7)	3001 (25.6)	<0.001
Location, N (%)				
- Right or transverse	7714 (44.2)	1902 (36.4)	5812 (47.6)	<0.001
- Left	5222 (29.9)	1582 (30.2)	3640 (29.8)	0.6
- Rectum	4510 (25.9)	1755 (33.4)	2755 (22.6)	<0.001
Tumor histological type, N (%)				
- Adenocarcinoma	17 229 (98.8)	5116 (97.5)	12 113 (99.2)	<0.001
- Squamous cell carcinoma	135 (0.8)	49 (0.9)	86 (0.7)	0.11
- No histology for palliative surgery	82 (0.5)	74 (1.4)	8 (0.1)	<0.001
SARS-CoV-2 pandemic period, N (%)	7572 (43.4)	2021 (38.6)	5551 (45.5)	<0.001
	(N=15 316)	(N=4791)	(N=10 525)	
Synchronous cancers, N (%)	538 (3.5)	136 (2.9)	402(3.8)	0.003
	(N=15 285)	(N=4723)	(N=10 562)	
Synchronous adenomas, N (%)	3294 (21.7)	1130 (24.2)	2164 (20.5)	<0.001
	(N=13 877)	(N=4174)	(N=9703)	
BMI, mean (SD)	25.6 (4.9)	25.6 (4.6)	25.6 (5.0)	0.82
BMI category, N (%)				
- <18	204 (1.5)	51 (1.2)	153 (1.6)	0.11
- ≥18 and <25	5796 (41.8)	1744 (41.7)	4052 (41.8)	0.98
- ≥25 and <30	5743 (41.4)	1721 (41.3)	4012 (41.5)	0.89
- ≥30 and <35	1629 (11.8)	504 (12.1)	1125 (11.6)	0.42
- ≥35	505 (3.6)	144 (3.7)	361 (3.5)	0.44

* T-test and Chi-squared test for continuous and categorical variables, respectively. BMI: body mass index; SD: Standard Deviation.

© 2022 Rottoli M et al. *JAMA Network Open.*

eTable 5. Comparison of the Clinical Characteristics and the Histological Variables According to the Secondary Outcome Stenotic Tumor

**Variables**	**Overall sample (N=17 938)**	**No stenotic Tumor (N=15 327)**	**Stenotic Tumor (N=2661)**	**p***
Mean age in years (SD)	70.6 (12.2)	70.4 (12.2)	71.2 (12.5)	0.002
Age class, N (%)				
- <60	3437 (19.2)	2945 (19.2)	492 (18.4)	0.66
- 60-69	3969 (22.1)	3456 (22.6)	513 (19.7)	0.001
- 70-79	5766 (32.1)	4946 (32.7)	820 (31.4)	0.38
- ≥80	4766 (26.6)	3980 (26.0)	786 (30.1)	<0.001
Male sex, N (%)	10 007 (55.8)	8552 (55.8)	1455 (55.7)	0.95
Asymptomatic, N (%)	3135 (17.6)	2948 (19.2)	205 (7.9)	<0.001
Positive fecal occult blood test screening, N (%)	4529 (26.4)	3979 (27.2)	550 (21.6)	<0.001
Location, N (%)				
- Right or transverse	7750 (43.2)	6978 (45.1)	772 (29.6)	<0.001
- Left	5253 (29.3)	3920 (25.6)	1333 (51.1)	<0.001
- Rectum	4935 (27.5)	4429 (28.9)	506 (19.4)	<0.001
Tumor histological type, N (%)				
- Adenocarcinoma	17 626 (98.3)	15 071 (98.3)	2555 (97.9)	0.09
- Squamous cell carcinoma	145 (0.8)	137 (0.9)	8 (0.3)	0.002
- No histology for palliative surgery	167 (0.9)	119 (0.8)	48 (1.8)	<0.001
SARS-CoV-2 pandemic period, N (%)	7769 (43.5)	6581 (43.0)	1215 (46.5)	0.001
	(N=15 710)	(N=13 211)	(N=2499)	
Synchronous cancers, N (%)	545 (3.5)	423 (3.2)	122 (4.9)	<0.001
	(N=15 684)	(N=13 115)	(N=2529)	
Synchronous adenomas, N (%)	3372 (21.5)	3061 (23.3)	311 (12.3)	<0.001
	(N=14 295)	(N=12 418)	(N=2103)	
BMI, mean (SD)	25.6 (4.8)	25.7 (4.8)	25.1 (4.9)	<0.001
BMI category, N (%)				
- <18	218 (1.5)	174 (1.4)	44 (2.1)	0.025
- ≥18 and <25	5976 (41.8)	4986 (40.9)	990 (47.1)	<0.001
- ≥25 and <30	5909 (41.3)	5121 (42.0)	788 (37.5)	<0.001
- ≥30 and <35	1680 (11.8)	212 (12.0)	1468 (10.1)	0.01
- ≥35	512 (3.6)	443 (3.6)	69 (3.3)	0.42

* T-test and Chi-squared test for continuous and categorical variables, respectively. BMI: body mass index; SD: Standard Deviation.

© 2022 Rottoli M et al. *JAMA Network Open.*

eTable 6. Comparison of the Clinical Characteristics and the Histological Variables According to the Secondary Outcome Urgent Surgery

Variables	Overall sample (N=17 938)	Non Urgent Surgery (N=15 913)	Urgent Surgery (N=2025)	p*
Mean age in years (SD)	70.6 (12.2)	73.3 (13.3)	70.2 (12.1)	<0.001
Age class, N (%)				
- <60	3437 (19.2)	3109 (19.5)	328 (16.2)	<0.001
- 60-69	3969 (22.1)	3628 (22.8)	341 (16.8)	<0.001
- 70-79	5766 (32.1)	5210 (32.7)	556 (27.5)	<0.001
- ≥80	4766 (26.6)	3966 (24.9)	800 (39.5)	<0.001
Male sex, N (%)	10 007 (55.8)	8973 (56.4)	1034 (51.1)	<0.001
Asymptomatic, N (%)	3153 (17.6)	3110 (19.5)	43 (2.1)	<0.001
Positive fecal occult blood test screening, N (%)	4529 (26.4)	4381 (28.7)	148 (7.7)	<0.001
Location, N (%)				
- Right or transverse	7750 (43.2)	6655 (41.8)	1095 (54.1)	<0.001
- Left	5253 (29.3)	4631 (29.1)	622 (30.7)	0.13
- Rectum	4935 (27.5)	4627 (29.1)	308 (15.2)	<0.001
Tumor histological type, N (%)				
- Adenocarcinoma	17 626 (98.3)	15 693 (98.6)	1933 (95.5)	<0.001
- Squamous cell carcinoma	145 (0.8)	130 (0.8)	15 (0.7)	0.72
- No histology for palliative surgery	167 (0.9)	90 (0.6)	77 (3.8)	<0.001
SARS-CoV-2 pandemic period, N (%)	7796 (43.5)	6847 (43.0)	949 (46.9)	0.001
	(N=15 710)	(N=14 814)	(N=896)	
Synchronous cancers, N (%)	545 (3.5)	511 (3.5)	34 (3.8)	0.58
	(N=15 684)	(N=14 801)	(N=883)	
Synchronous adenomas, N (%)	3372 (21.5)	3249 (22.0)	123 (13.9)	<0.001
	(N=14 295)	(N=12 925)	(N=1370)	
BMI, mean (SD)	25.6 (4.8)	25.6 (4.8)	25.3 (5.3)	0.012
BMI category, N (%)				
- <18	218 (1.5)	184 (1.4)	34 (1.2)	0.043
- ≥18 and <25,	5976 (41.8)	5405 (41.8)	571 (28.1)	<0.001
- ≥25 and <30	5909 (41.3)	5324 (41.2)	585 (29.0)	<0.001
- ≥30 and <35,	1680 (11.8)	1546 (12.0)	134 (6.7)	<0.001
- ≥35	512 (3.6)	466 (3.6)	46 (3.4)	<0.001

* T-test and Chi-squared test for continuous and categorical variables, respectively. BMI: body mass index; SD: Standard Deviation.

© 2022 Rottoli M et al. *JAMA Network Open.*

eTable 7. Comparison of the Clinical Characteristics and the Histological Variables According to the Secondary Outcome Palliative Surgery

Variables	Overall sample (N=17 938)	Curative surgery (N=16 559)	Palliative surgery (N=1379)	p*
Mean age in years (SD)	70.6 (12.2)	70.4 (12.2)	72.4 (12.9)	<0.001
Age class, N (%)				
- <60	3437 (19.2)	3199 (19.3)	238 (17.3)	0.062
- 60-69	3969 (22.1)	3715 (22.4)	254 (18.4)	0.001
- 70-79	5766 (32.1)	5357 (32.4)	409 (29.7)	0.040
- ≥80,	4766 (26.6)	4288 (25.9)	478 (34.7)	<0.001
Male sex, N (%)	10 007 (55.8)	9244 (55.8)	763 (55.3)	0.72
Asymptomatic, N (%)	3153 (17.6)	3063 (18.5)	90 (6.5)	<0.001
Positive fecal occult blood test screening, N (%)	4529 (26.4)	4360 (27.5)	169 (12.8)	<0.001
Location, N (%)				
- Right or transverse	7750 (43.2)	7151 (43.2)	599 (43.4)	0.86
- Left	5253 (29.3)	4829 (29.2)	424 (30.8)	0.21
- Rectum	4935 (27.5)	4579 (27.7)	356 (25.8)	0.14
Tumor histological type, N (%)	(N=17 771)	(N=16 559)	(N=1212)	
- Adenocarcinoma	17 626 (99.2)	16 437 (99.3)	1189 (98.1)	<0.001
- Squamous cell carcinoma	145 (0.8)	122 (0.7)	23 (1.9)	<0.001
SARS-CoV-2 pandemic period, N (%)	7796 (43.5)	7152 (43.2)	644 (46.7)	0.012
	(N=15 710)	(N=14 7254)	(N=985)	
Synchronous cancers, N (%)	545 (3.5)	501 (3.4)	44 (4.5)	0.08
	(N=15 684)	(N=14 719)	(N=965)	
Synchronous adenomas, N (%)	3372 (21.5)	3246 (22.1)	126 (13.1)	<0.001
	(N=14 295)	(N=13 256)	(N=1039)	
BMI, mean (SD)	25.6 (4.8)	25.7 (4.8)	24.5 (5.0)	<0.001
BMI category, N (%)				
- <18	218 (1.5)	176 (1.3)	42 (4.0)	<0.001
- ≥18 and <25	5976 (41.8)	5473 (41.3)	503 (48.4)	0.01
- ≥25 and <30	5909 (41.3)	5530 (41.7)	379 (36.5)	<0.001
- ≥30 and <35	1680 (11.8)	1592 (12.0)	88 (8.5)	<0.001
- ≥35	512 (3.6)	485 (3.7)	27 (2.6)	0.002

* T-test and Chi-squared test for continuous and categorical variables, respectively. BMI: body mass index; SD: Standard Deviation.

© 2022 Rottoli M et al. *JAMA Network Open.*
