# Supplementary material for: Colorectal Cancer Stage at Diagnosis Before vs During the COVID-19 Pandemic in Italy
Source: JAMA Netw Open. 2022 Nov 21;5(11):e2243119. doi: 10.1001/jamanetworkopen.2022.43119 (PMC9679872; doi:10.1001/jamanetworkopen.2022.43119)
Supplement: Supplement 2. — Nonauthor Collaborators. COVID–Colorectal Cancer (CRC) Study Group [file jamanetwopen-e2243119-s002.pdf]

***Group Name(s): COVID–Colorectal Cancer (CRC) Study Group**

***First Name and Middle Initial(s)**	***Last Name**	***Suffix (eg, Jr, III)**	Academic Degrees	Institution	Location (city, state/province, country)	Role or Contribution, eg, chair, principal investigator	Group (if more than 1 Group listed in the byline) and/or Subgroup (eg, Steering Committee)
Angela	Romano			Surgery of the AlimentaryTract, IRCCS Azienda Ospedaliero-Univers	Bologna, Italy	Data Collector	
Angela	Belvedere			Surgery of the AlimentaryTract, IRCCS Azienda Ospedaliero-Univers	Bologna, Italy	Data Collector	
Antonio	Lanci Lanci			Surgery of the AlimentaryTract, IRCCS Azienda Ospedaliero-Univers	Bologna, Italy	Data Collector	
Daniele	Parlanti			Surgery of the AlimentaryTract, IRCCS Azienda Ospedaliero-Univers	Bologna, Italy	Data Collector	
Gabriele	Vago			Surgery of the AlimentaryTract, IRCCS Azienda Ospedaliero-Univers	Bologna, Italy	Data Collector	
Paola	Pezzuto			Surgery of the AlimentaryTract, IRCCS Azienda Ospedaliero-Univers	Bologna, Italy	Data Collector	
Anna	Canavese			Surgery of the AlimentaryTract, IRCCS Azienda Ospedaliero-Univers	Bologna, Italy	Data Collector	
Gerti	Dajti			Surgery of the AlimentaryTract, IRCCS Azienda Ospedaliero-Univers	Bologna, Italy	Data Collector	
Stefano	Cardelli			Surgery of the AlimentaryTract, IRCCS Azienda Ospedaliero-Univers	Bologna, Italy	Data Collector	
Caterina	Catalioto			Surgery of the AlimentaryTract, IRCCS Azienda Ospedaliero-Univers	Bologna, Italy	Data Collector	
Iris S	Russo			Surgery of the AlimentaryTract, IRCCS Azienda Ospedaliero-Univers	Bologna, Italy	Data Collector	
Tommaso	Violante			Surgery of the AlimentaryTract, IRCCS Azienda Ospedaliero-Univers	Bologna, Italy	Data Collector	
Daniele	Morezzi			Surgery of the AlimentaryTract, IRCCS Azienda Ospedaliero-Univers	Bologna, Italy	Data Collector	
Ludovica	Maurino			Surgery of the AlimentaryTract, IRCCS Azienda Ospedaliero-Univers	Bologna, Italy	Data Collector	
Eleonora	Filippone			Surgery of the AlimentaryTract, IRCCS Azienda Ospedaliero-Univers	Bologna, Italy	Data Collector	
Dajana	Cuicchi			Surgery of the AlimentaryTract, IRCCS Azienda Ospedaliero-Univers	Bologna, Italy	Data Collector	
Paolo	Bernante			Surgery of the AlimentaryTract, IRCCS Azienda Ospedaliero-Universitaria di Bologna, Bologna, Italy; Dep		Data Collector	
Elio	Jovine			Chirurgia A e d'Urgenza IRCCS AOU c/o OM, IRCCS Azienda Ospedal	Bologna, Italy	Principal Investigator	
Raffaele	Lombardi			Chirurgia A e d'Urgenza IRCCS AOU c/o OM, IRCCS Azienda Ospedal	Bologna, Italy	Data Collector	
Michele	Masetti			Chirurgia A e d'Urgenza IRCCS AOU c/o OM, IRCCS Azienda Ospedal	Bologna, Italy	Data Collector	
Chiara	Cipressi			Chirurgia A e d'Urgenza IRCCS AOU c/o OM, IRCCS Azienda Ospedal	Bologna, Italy	Data Collector	
Maria F	Offi			Chirurgia A e d'Urgenza IRCCS AOU c/o OM, IRCCS Azienda Ospedal	Bologna, Italy	Data Collector	
Cristina	Larotonda			Chirurgia A e d'Urgenza IRCCS AOU c/o OM, IRCCS Azienda Ospedal	Bologna, Italy	Data Collector	
Silvana B	Puglisi			Chirurgia A e d'Urgenza IRCCS AOU c/o OM, IRCCS Azienda Ospedal	Bologna, Italy	Data Validator	
Augusto	Barbosa			Fondazione Poliambulanza Brescia	Brescia, Italy	Principal Investigator; Data	
Roberto	Vaiana			Fondazione Poliambulanza Brescia	Brescia, Italy	Data Collector	
Paolo M	Bianchi			Fondazione Poliambulanza Brescia	Brescia, Italy	Data Collector	
Carlo	Tonti			Fondazione Poliambulanza Brescia	Brescia, Italy	Data Collector	
Claudio	Codignola			Fondazione Poliambulanza Brescia	Brescia, Italy	Data Collector	
Luigi	Zorcolo			Unità operativa di Chirurgia Coloproctologica - AOU Cagliari	Cagliari, Italy	Principal Investigator	
Angelo	Restivo			Unità operativa di Chirurgia Coloproctologica - AOU Cagliari	Cagliari, Italy	Data Collector	
Simona	Deidda			Unità operativa di Chirurgia Coloproctologica - AOU Cagliari	Cagliari, Italy	Data Collector	
Marcello E	Marchetti			Unità operativa di Chirurgia Coloproctologica - AOU Cagliari	Cagliari, Italy	Data Collector	
Luca	Ippolito			Unità operativa di Chirurgia Coloproctologica - AOU Cagliari	Cagliari, Italy	Data Validator	
Gaya	Spolverato			Department of SurgicalOncological and Gastroenterological Science	Padova, Italy	Principal Investigator	
Salvatore	Pucciarelli			Department of SurgicalOncological and Gastroenterological Science	Padova, Italy	Data Collector	
Francesco	Marchegiani			Department of SurgicalOncological and Gastroenterological Science	Padova, Italy	Data Collector	
Giacomo	Ghio			Department of SurgicalOncological and Gastroenterological Science	Padova, Italy	Data Collector	
Gaya	Zagolin			Department of SurgicalOncological and Gastroenterological Science	Padova, Italy	Data Collector	
Dajana	Glavas			Department of SurgicalOncological and Gastroenterological Science	Padova, Italy	Data Collector	
Monica	Tomassi			Department of SurgicalOncological and Gastroenterological Science	Padova, Italy	Data Validator	
Riccardo	Rosati			Gastrointestinal Surgery Division, IRCCS San Raffaele Hospital, Milan	Milano, Italy	Principal Investigator	
Ugo	Elmore			Gastrointestinal Surgery Division, IRCCS San Raffaele Hospital, Milan	Milano, Italy	Data Collector	
Lorenzo	Gozzini			Gastrointestinal Surgery Division, IRCCS San Raffaele Hospital, Milan	Milano, Italy	Data Collector	
Riccardo	Calef			Gastrointestinal Surgery Division, IRCCS San Raffaele Hospital, Milan	Milano, Italy	Data Validator	
Francesco	Puccetti			Gastrointestinal Surgery Division, IRCCS San Raffaele Hospital, Milan	Milano, Italy	Data Collector	

Supplemental Online Content: Nonauthor Collaborators*First name, last name, and suffix (if applicable) are required and will appear in PubMed.

*First Name and Middle Initial(s)	*Last Name	*Suffix (eg, Jr, III)	Academic Degrees	Institution	Location (city, state/province, country)	Role or Contribution, eg, chair, principal investigator	Group (if more than 1 Group listed in the byline) and/or Subgroup (eg, Steering Committee)
Andrea	Cossu			Gastrointestinal Surgery Division, IRCCS San Raffaele Hospital, Milan	Milano, Italy	Data Collector	
Andrea	Vignali			Gastrointestinal Surgery Division, IRCCS San Raffaele Hospital, Milan	Milano, Italy	Data Collector	
Mario	Morino			AOU Città della Salute e della Scienza, Turin, Italy	Torino, Italy	Principal Investigator	
Marco E	Allaix			AOU Città della Salute e della Scienza, Turin, Italy	Torino, Italy	Data Collector	
Gaspare	Cannata			AOU Città della Salute e della Scienza, Turin, Italy	Torino, Italy	Data Collector	
Erica	Lombardi			AOU Città della Salute e della Scienza, Turin, Italy	Torino, Italy	Data Validator	
Carlo A	Ammirati			AOU Città della Salute e della Scienza, Turin, Italy	Torino, Italy	Data Collector	
Chiara	Piceni			AOU Città della Salute e della Scienza, Turin, Italy	Torino, Italy	Data Collector	
Piero	Buccianti			Azienda Ospedaliero-Universitaria Pisana	Pisa, Italy	Principal Investigator	
Riccardo	Balestri			Azienda Ospedaliero-Universitaria Pisana	Pisa, Italy	Data Collector	
Marco	Puccini			Azienda Ospedaliero-Universitaria Pisana	Pisa, Italy	Data Collector	
Daniele	Pezzati			Azienda Ospedaliero-Universitaria Pisana	Pisa, Italy	Data Validator	
Roberto	d'Ischia			Azienda Ospedaliero-Universitaria Pisana	Pisa, Italy	Data Collector	
Vito F	Asta			Azienda Ospedaliero-Universitaria Pisana	Pisa, Italy	Data Collector	
Benedetta	Sargenti			Azienda Ospedaliero-Universitaria Pisana	Pisa, Italy	Data Collector	
Giacomo	Taddei			Azienda Ospedaliero-Universitaria Pisana	Pisa, Italy	Data Collector	
Federica	Bonari			Azienda Ospedaliero-Universitaria Pisana	Pisa, Italy	Data Collector	
Giulia	Boni			Azienda Ospedaliero-Universitaria Pisana	Pisa, Italy	Data Collector	
Alessandro	Ferrero			Azienda Sanitaria Ospedaliera Ordine Mauriziano Umberto I°, Torin	Torino, Italy	Principal Investigator	
Michela	Mineccia			Azienda Sanitaria Ospedaliera Ordine Mauriziano Umberto I°, Torin	Torino, Italy	Data Collector	
Federica	Gonella			Azienda Sanitaria Ospedaliera Ordine Mauriziano Umberto I°, Torin	Torino, Italy	Data Collector	
Marco	Palisi			Azienda Sanitaria Ospedaliera Ordine Mauriziano Umberto I°, Torin	Torino, Italy	Data Validator	
Francesco	Danese			Azienda Sanitaria Ospedaliera Ordine Mauriziano Umberto I°, Torin	Torino, Italy	Data Collector	
Valeria	Cherubini			Azienda Sanitaria Ospedaliera Ordine Mauriziano Umberto I°, Torin	Torino, Italy	Data Collector	
Serena	Perotti			Azienda Sanitaria Ospedaliera Ordine Mauriziano Umberto I°, Torin	Torino, Italy	Data Collector	
Michele	Carvello			IRCCS Humanitas Research Hospital, Rozzano, Milan, Italy	Rozzano, Milano, Italy	Principal Investigator	
Fabio	Carbone			IRCCS Humanitas Research Hospital, Rozzano, Milan, Italy	Rozzano, Milano, Italy	Data Collector	
Antonio	Luberto			IRCCS Humanitas Research Hospital, Rozzano, Milan, Italy	Rozzano, Milano, Italy	Data Collector	
Eleonora	Calafiore			IRCCS Humanitas Research Hospital, Rozzano, Milan, Italy	Rozzano, Milano, Italy	Data Collector	
Francesca	De Lucia			IRCCS Humanitas Research Hospital, Rozzano, Milan, Italy	Rozzano, Milano, Italy	Data Collector	
Matteo	Sacchi			IRCCS Humanitas Research Hospital, Rozzano, Milan, Italy	Rozzano, Milano, Italy	Data Validator	
Diego	Sasia			Santa Croce and Carle Hospital, Cuneo	Cuneo, Italy	Principal Investigator	
Maria C	Giuffrida			Santa Croce and Carle Hospital, Cuneo	Cuneo, Italy	Data Collector	
Edoardo	Ballauri			Santa Croce and Carle Hospital, Cuneo	Cuneo, Italy	Data Collector	
Mathieu	Cardile			Santa Croce and Carle Hospital, Cuneo	Cuneo, Italy	Data Collector	
Serena	Armentano			Santa Croce and Carle Hospital, Cuneo	Cuneo, Italy	Data Collector	
Elsa	Beltrami			Santa Croce and Carle Hospital, Cuneo	Cuneo, Italy	Data Collector	
Gabriele	Preve			Santa Croce and Carle Hospital, Cuneo	Cuneo, Italy	Data Collector	
Barbara	Vercellone			Santa Croce and Carle Hospital, Cuneo	Cuneo, Italy	Data Validator	
Marta	Mozzon			UO Chirurgia generale, Azienda Ospedaliera Universitaria Friuli Cen	Udine, Italy	Principal Investigator; Data	
Cristina	Folliero			UO Chirurgia generale, Azienda Ospedaliera Universitaria Friuli Cen	Udine, Italy	Data Collector	
Chiara	Lirusso			UO Chirurgia generale, Azienda Ospedaliera Universitaria Friuli Cen	Udine, Italy	Data Collector	
Massimo	Vecchiato			UO Chirurgia generale, Azienda Ospedaliera Universitaria Friuli Cen	Udine, Italy	Data Collector	
Antonio	Ziccarelli			UO Chirurgia generale, Azienda Ospedaliera Universitaria Friuli Cen	Udine, Italy	Data Collector	
Davide	Gattesco			UO Chirurgia generale, Azienda Ospedaliera Universitaria Friuli Cen	Udine, Italy	Data Collector	
Luisa	Moretti			UO Chirurgia generale, Azienda Ospedaliera Universitaria Friuli Cen	Udine, Italy	Data Collector	
Sara	Crestale			UO Chirurgia generale, Azienda Ospedaliera Universitaria Friuli Cen	Udine, Italy	Data Collector	
Filippo	Banchini			UO Chirurgia Generale Vascolare di Piacenza	Piacenza, Italy	Principal Investigator; Data	

***First Name and Middle Initial(s)**	***Last Name**	***Suffix (eg, Jr, III)**	Academic Degrees	Institution	Location (city, state/province, country)	Role or Contribution, eg, chair, principal investigator	Group (if more than 1 Group listed in the byline) and/or Subgroup (eg, Steering Committee)
Patrizio	Capelli			UO Chirurgia Generale Vascolare di Piacenza	Piacenza, Italy	Data Collector	
Andrea	Romboli			UO Chirurgia Generale Vascolare di Piacenza	Piacenza, Italy	Data Collector	
Gerardo	Palmieri			UO Chirurgia Generale Vascolare di Piacenza	Piacenza, Italy	Data Collector	
Luigi	Conti			UO Chirurgia Generale Vascolare di Piacenza	Piacenza, Italy	Data Collector	
Nicholas	Rizzi			UO Chirurgia Generale Vascolare di Piacenza	Piacenza, Italy	Data Collector	
Deborah	Bonfili			Dipartimento di Chirurgia, Università degli Studi di Parma	Piacenza, Italy	Data Collector	
Nicolò	de Manzini			General surgery department, University Hospital of Trieste	Trieste, Italy	Principal Investigator	
Paola	Germani			General surgery department, University Hospital of Trieste	Trieste, Italy	Data Collector	
Edoardo	Osenda			General surgery department, University Hospital of Trieste	Trieste, Italy	Data Collector	
Sara	Cortinovis			General surgery department, University Hospital of Trieste	Trieste, Italy	Data Collector	
Carlotta	Giunta			General surgery department, University Hospital of Trieste	Trieste, Italy	Data Collector	
Stefano	Fracon			General surgery department, University Hospital of Trieste	Trieste, Italy	Data Collector	
Hussein	Abdallah			General surgery department, University Hospital of Trieste	Trieste, Italy	Data Collector	
Selene	Bogoni			General surgery department, University Hospital of Trieste	Trieste, Italy	Data Validator	
Nazario	Portolani			U.O. Chirurgia Generale 3 - ASST Spedali Civili Brescia, Università di	Brescia, Italy	Principal Investigator	
Riccardo	Nascimbeni			U.O. Chirurgia Generale 3 - ASST Spedali Civili Brescia, Università di	Brescia, Italy	Data Validator	
Sarah	Molfino			U.O. Chirurgia Generale 3 - ASST Spedali Civili Brescia, Università di	Brescia, Italy	Data Collector	
Guido A M	Tiberio			U.O. Chirurgia Generale 3 - ASST Spedali Civili Brescia, Università di	Brescia, Italy	Data Collector	
Ilenia	Garosio			U.O. Chirurgia Generale 3 - ASST Spedali Civili Brescia, Università di	Brescia, Italy	Data Collector	
Giulia	Lamperti			U.O. Chirurgia Generale 3 - ASST Spedali Civili Brescia, Università di	Brescia, Italy	Data Collector	
Diego	Rigosa			U.O. Chirurgia Generale 3 - ASST Spedali Civili Brescia, Università di	Brescia, Italy	Data Collector	
Giorgio	Ercolani			Chirurgia generale e TOA, Ospedale Morgagni-Pierantoni, Forlì	Forlì, Italy	Principal Investigator	
Leonardo	Solaini			Chirurgia generale e TOA, Ospedale Morgagni-Pierantoni, Forlì	Forlì, Italy	Data Collector	
Davide	Cavaliere			Chirurgia generale e TOA, Ospedale Morgagni-Pierantoni, Forlì	Forlì, Italy	Data Collector	
Andrea	Avanzolini			Chirurgia generale e TOA, Ospedale Morgagni-Pierantoni, Forlì	Forlì, Italy	Data Collector	
Fabrizio	D'Acapito			Chirurgia generale e TOA, Ospedale Morgagni-Pierantoni, Forlì	Forlì, Italy	Data Collector	
Leonardo L	Chiarella			Chirurgia generale e TOA, Ospedale Morgagni-Pierantoni, Forlì	Forlì, Italy	Data Collector	
Daniela	Di Pietrantonio			Chirurgia generale e TOA, Ospedale Morgagni-Pierantoni, Forlì	Forlì, Italy	Data Collector	
Domenico	Annunziata			Chirurgia generale e TOA, Ospedale Morgagni-Pierantoni, Forlì	Forlì, Italy	Data Validator	
Roberta	Piccolo			U.O. Chirurgia Generale Ospedale di Latisana-Palmanova, Azienda (	Latisana, Udine, Italy	Principal Investigator; Data	
Mario	Sorrentino			U.O. Chirurgia Generale Ospedale di Latisana-Palmanova, Azienda (	Latisana, Udine, Italy	Data Collector	
Mauro	Pansini			U.O. Chirurgia Generale Ospedale di Latisana-Palmanova, Azienda (	Latisana, Udine, Italy	Data Collector	
Alessandro	Cojutti			U.O. Chirurgia Generale Ospedale di Latisana-Palmanova, Azienda (	Latisana, Udine, Italy	Data Collector	
Michele	Graziano			U.O. Chirurgia Generale Ospedale di Latisana-Palmanova, Azienda (	Latisana, Udine, Italy	Data Collector	
Francesco	Callegari			U.O. Chirurgia Generale Ospedale di Latisana-Palmanova, Azienda (	Latisana, Udine, Italy	Data Collector	
Laura	Balzarotti			Ospedale civile “G. Fornaroli”, Magenta	Magenta, Milano, Italy	Principal Investigator; Data	
Vitale R	Dameno			Ospedale civile “G. Fornaroli”, Magenta	Magenta, Milano, Italy	Data Collector	
Antonio	Cattaneo			Ospedale civile “G. Fornaroli”, Magenta	Magenta, Milano, Italy	Data Collector	
Giuliano	Santolamazza			Ospedale civile “G. Fornaroli”, Magenta	Magenta, Milano, Italy	Data Collector	
Caterina	Altieri			Ospedale civile “G. Fornaroli”, Magenta	Magenta, Milano, Italy	Data Collector	
Riccardo	Magarini			Ospedale civile “G. Fornaroli”, Magenta	Magenta, Milano, Italy	Data Collector	
Andrea	Pietrabissa			Department of Surgery, University of Pavia and Fondazione IRCCS P	Pavia, Italy	Principal Investigator	
Tommaso	Dominioni			Department of Surgery, University of Pavia and Fondazione IRCCS P	Pavia, Italy	Data Collector	
Luigi	Pugliese			Department of Surgery, University of Pavia and Fondazione IRCCS P	Pavia, Italy	Data Collector	
Andrea	Peri			Department of Surgery, University of Pavia and Fondazione IRCCS P	Pavia, Italy	Data Collector	
Marta	Botti			Department of Surgery, University of Pavia and Fondazione IRCCS P	Pavia, Italy	Data Collector	
Benedetta	Sargenti			Department of Surgery, University of Pavia and Fondazione IRCCS P	Pavia, Italy	Data Collector	
Francesco	Salvetti			Department of Surgery, University of Pavia and Fondazione IRCCS P	Pavia, Italy	Data Validator	

Supplemental Online Content: Nonauthor Collaborators*First name, last name, and suffix (if applicable) are required and will appear in PubMed.

*First Name and Middle Initial(s)	*Last Name	*Suffix (eg, Jr, III)	Academic Degrees	Institution	Location (city, state/province, country)	Role or Contribution, eg, chair, principal investigator	Group (if more than 1 Group listed in the byline) and/or Subgroup (eg, Steering Committee)
Elisa	Cassinotti			Fondazione IRCCS Ca' Granda Ospedale Maggiore Policlinico - Milan	Milano, Italy	Principal Investigator; Data	
Ludovica	Baldari			Fondazione IRCCS Ca' Granda Ospedale Maggiore Policlinico - Milan	Milano, Italy	Data Collector	
Luigi	Boni			Fondazione IRCCS Ca' Granda Ospedale Maggiore Policlinico - Milan	Milano, Italy	Data Collector	
Valentina	Messina			Fondazione IRCCS Ca' Granda Ospedale Maggiore Policlinico - Milan	Milano, Italy	Data Collector	
Vera	D'Abrosca			Fondazione IRCCS Ca' Granda Ospedale Maggiore Policlinico - Milan	Milano, Italy	Data Collector	
Pasquale	Cianci			UOC Chirurgia Generale, Ospedale Lorenzo Bonomo, Andria	Andria, BAT, Italy	Principal Investigator; Data	
Rocco	Tumolo			UOC Chirurgia Generale, Ospedale Lorenzo Bonomo, Andria	Andria, BAT, Italy	Data Collector	
Domenico	Gattulli			UOC Chirurgia Generale, Ospedale Lorenzo Bonomo, Andria	Andria, BAT, Italy	Data Collector	
Enrico	Restini			UOC Chirurgia Generale, Ospedale Lorenzo Bonomo, Andria	Andria, BAT, Italy	Data Collector	
Marina	Minafra			UOC Chirurgia Generale, Ospedale Lorenzo Bonomo, Andria	Andria, BAT, Italy	Data Collector	
Maria G	Sederino			UOC Chirurgia Generale, Ospedale Lorenzo Bonomo, Andria	Andria, BAT, Italy	Data Collector	
Bernardino	Bottalico			UOC Chirurgia Generale, Ospedale Lorenzo Bonomo, Andria	Andria, BAT, Italy	Data Collector	
Pierluigi	Pilati			Unit of Surgical Oncology of Digestive Tract, Veneto Institute of Onc	Castelfranco Veneto, Treviso, Italy	Principal Investigator	
Boris	Franzato			Unit of Surgical Oncology of Digestive Tract, Veneto Institute of Onc	Castelfranco Veneto, Treviso, Italy	Data Collector	
Genny	Mattara			Unit of Surgical Oncology of Digestive Tract, Veneto Institute of Onc	Castelfranco Veneto, Treviso, Italy	Data Collector	
Ottavia	De Simoni			Unit of Surgical Oncology of Digestive Tract, Veneto Institute of Onc	Castelfranco Veneto, Treviso, Italy	Data Collector	
Andrea	Barina			Unit of Surgical Oncology of Digestive Tract, Veneto Institute of Onc	Castelfranco Veneto, Treviso, Italy	Data Collector	
Marco	Tonello			Unit of Surgical Oncology of Digestive Tract, Veneto Institute of Onc	Castelfranco Veneto, Treviso, Italy	Data Validator	
Andrea	Muratore			Chirurgia Generale Ospedale E. Agnelli, Pinerolo	Pinerolo, Torino, Italy	Principal Investigator	
Marcello	Calabrò			Chirurgia Generale Ospedale E. Agnelli, Pinerolo	Pinerolo, Torino, Italy	Data Validator	
Nicoletta S	Federico Pipitone			Chirurgia Generale Ospedale E. Agnelli, Pinerolo	Pinerolo, Torino, Italy	Data Collector	
Bruno	Cuzzola			Chirurgia Generale Ospedale E. Agnelli, Pinerolo	Pinerolo, Torino, Italy	Data Collector	
Elena	Herranz van Nood			Chirurgia Generale Ospedale E. Agnelli, Pinerolo	Pinerolo, Torino, Italy	Data Collector	
Nicola	Passuello			U.O.C. Chirurgia Generale OSA, io DIDAS Chirurgia, Azienda Ospeda	Padova, Italy	Principal Investigator; Data	
Alvise	Frasson			U.O.C. Chirurgia Generale OSA, io DIDAS Chirurgia, Azienda Ospeda	Padova, Italy	Data Collector	
Enzo	Mammano			U.O.C. Chirurgia Generale OSA, io DIDAS Chirurgia, Azienda Ospeda	Padova, Italy	Data Collector	
Luca	Faccio			U.O.C. Chirurgia Generale OSA, io DIDAS Chirurgia, Azienda Ospeda	Padova, Italy	Data Collector	
Fabrizio	Vittadello			U.O.C. Chirurgia Generale OSA, io DIDAS Chirurgia, Azienda Ospeda	Padova, Italy	Data Collector	
Alice	Bressan			U.O.C. Chirurgia Generale OSA, io DIDAS Chirurgia, Azienda Ospeda	Padova, Italy	Data Collector	
Giacomo	Sarzo			U.O.C. Chirurgia Generale OSA, io DIDAS Chirurgia, Azienda Ospeda	Padova, Italy	Data Collector	
Nicolò	Tamini			ASST Monza - Ospedale San Gerardo	Monza, Italy	Principal Investigator; Data	
Massimo	Oldani			ASST Monza - Ospedale San Gerardo	Monza, Italy	Data Collector	
Luca	Cigagna			ASST Monza - Ospedale San Gerardo	Monza, Italy	Data Collector	
Francesca	Carissimi			ASST Monza - Ospedale San Gerardo	Monza, Italy	Data Collector	
Giulia	De Carlo			ASST Monza - Ospedale San Gerardo	Monza, Italy	Data Collector	
Edoardo	Baccalini			ASST Monza - Ospedale San Gerardo	Monza, Italy	Data Collector	
Luca	Nespoli			ASST Monza - Ospedale San Gerardo	Monza, Italy	Data Collector	
Alessio	Giordano			UO di Chirurgia Generale, Nuovo Ospedale “S.Stefano”, Azienda AS	Prato, Italy	Principal Investigator	
Stefano	Cantafio			UO di Chirurgia Generale, Nuovo Ospedale “S.Stefano”, Azienda AS	Prato, Italy	Data Collector	
Lucrezia	Grifoni			UO di Chirurgia Generale, Nuovo Ospedale “S.Stefano”, Azienda AS	Prato, Italy	Data Collector	
Davide	Matani			UO di Chirurgia Generale, Nuovo Ospedale “S.Stefano”, Azienda AS	Prato, Italy	Data Collector	
Serena	Livi			UO di Chirurgia Generale, Nuovo Ospedale “S.Stefano”, Azienda AS	Prato, Italy	Data Validator	
Daniele	Delogu			Patologia Chirurgica AOU, Sassari	Sassari, Italy	Principal Investigator	
Fabrizio	Scognamillo			Patologia Chirurgica AOU, Sassari	Sassari, Italy	Data Collector	
Antonio	Marrosu			Patologia Chirurgica AOU, Sassari	Sassari, Italy	Data Collector	
Luca	Guerrini			Patologia Chirurgica AOU, Sassari	Sassari, Italy	Data Validator	
Giampaolo	Ugolini			UO Chirurgia Generale di Ravenna-Faenza, AUSL Romagna	Faenza, Ravenna, Italy	Principal Investigator	

*First Name and Middle Initial(s)	*Last Name	*Suffix (eg, Jr, III)	Academic Degrees	Institution	Location (city, state/province, country)	Role or Contribution, eg, chair, principal investigator	Group (if more than 1 Group listed in the byline) and/or Subgroup (eg, Steering Committee)
Federico	Ghignone			UO Chirurgia Generale di Ravenna-Faenza, AUSL Romagna	Faenza, Ravenna, Italy	Data Collector	
Giacomo	Frascaroli			UO Chirurgia Generale di Ravenna-Faenza, AUSL Romagna	Faenza, Ravenna, Italy	Data Collector	
Nicola	Albertini			UO Chirurgia Generale di Ravenna-Faenza, AUSL Romagna	Faenza, Ravenna, Italy	Data Validator	
Davide	Zattoni			UO Chirurgia Generale di Ravenna-Faenza, AUSL Romagna	Faenza, Ravenna, Italy	Data Collector	
Giovanni	Taffurelli			UO Chirurgia Generale di Ravenna-Faenza, AUSL Romagna	Faenza, Ravenna, Italy	Data Collector	
Isacco	Montroni			UO Chirurgia Generale di Ravenna-Faenza, AUSL Romagna	Faenza, Ravenna, Italy	Data Collector	
Francesco	Colombo			Division of General Surgery - L. Sacco University Hospital- Milano	Milano, Italy	Principal Investigator	
Piergiorgio	Danelli			Division of General Surgery - L. Sacco University Hospital- Milano	Milano, Italy	Data Collector	
Andrea	Bondurri			Division of General Surgery - L. Sacco University Hospital- Milano	Milano, Italy	Data Collector	
Anna	Maffioli			Division of General Surgery - L. Sacco University Hospital- Milano	Milano, Italy	Data Collector	
Alessandro	Bonomi			Division of General Surgery - L. Sacco University Hospital- Milano	Milano, Italy	Data Collector	
Isabella	Pezzoli			Division of General Surgery - L. Sacco University Hospital- Milano	Milano, Italy	Data Collector	
Francesco	Cammarata			Division of General Surgery - L. Sacco University Hospital- Milano	Milano, Italy	Data Validator	
Orlando	Goletti			Chirurgia Generale Humanitas Gavazzeni Bergamo, Italy	Bergamo, Italy	Principal Investigator	
Mattia	Molteni			Chirurgia Generale Humanitas Gavazzeni Bergamo, Italy	Bergamo, Italy	Data Validator	
Alberto	Assisi			Chirurgia Generale Humanitas Gavazzeni Bergamo, Italy	Bergamo, Italy	Data Collector	
Giorgio	Quartierini			Chirurgia Generale Humanitas Gavazzeni Bergamo, Italy	Bergamo, Italy	Data Collector	
Corrado	Da Lio			Department of General Surgery, Mirano Hospital, Venice	Mirano, Venezia, Italy	Principal Investigator; Data	
Daunia	Verdi			Department of General Surgery, Mirano Hospital, Venice	Mirano, Venezia, Italy	Data Collector	
Isabella	Mondi			Department of General Surgery, Mirano Hospital, Venice	Mirano, Venezia, Italy	Data Collector	
Claudia	Peluso			Department of General Surgery, Mirano Hospital, Venice	Mirano, Venezia, Italy	Data Collector	
Lorenzo	Macchi			Department of General Surgery, Mirano Hospital, Venice	Mirano, Venezia, Italy	Data Collector	
Marta	Tanzanu			Chirurgia d'Urgenza, Santa Maria delle Croci - Ravenna	Ravenna, Italy	Principal Investigator; Data	
Federico	Zanzi			Chirurgia d'Urgenza, Santa Maria delle Croci - Ravenna	Ravenna, Italy	Data Collector	
Sara	Pellegrini			Chirurgia d'Urgenza, Santa Maria delle Croci - Ravenna	Ravenna, Italy	Data Collector	
Jacopo	Andreuccetti			General Surgery 2 , ASST Spedali Civili of Brescia	Brescia, Italy	Principal Investigator	
Rossella	D'Alessio			General Surgery 2 , ASST Spedali Civili of Brescia	Brescia, Italy	Data Validator	
Giusto	Pignata			General Surgery 2 , ASST Spedali Civili of Brescia	Brescia, Italy	Data Collector	
Michele	De Capua			General Surgery 2 , ASST Spedali Civili of Brescia	Brescia, Italy	Data Collector	
Ilaria	Canfora			General Surgery 2 , ASST Spedali Civili of Brescia	Brescia, Italy	Data Collector	
Luca	Ottaviani			General Surgery 2 , ASST Spedali Civili of Brescia	Brescia, Italy	Data Collector	
Pasquale	Lepiane			Ospedale San Paolo, Civitavecchia, Roma	Civitavecchia, Roma, Italy	Principal Investigator	
Andrea	Balla			Ospedale San Paolo, Civitavecchia, Roma	Civitavecchia, Roma, Italy	Data Collector	
Antonio	De Carlo			Ospedale San Paolo, Civitavecchia, Roma	Civitavecchia, Roma, Italy	Data Collector	
Federica	Saraceno			Ospedale San Paolo, Civitavecchia, Roma	Civitavecchia, Roma, Italy	Data Validator	
Rosa	Scaramuzzo			Ospedale San Paolo, Civitavecchia, Roma	Civitavecchia, Roma, Italy	Data Collector	
Anna	Guida			Ospedale San Paolo, Civitavecchia, Roma	Civitavecchia, Roma, Italy	Data Collector	
Daniele	Aguzzi			Ospedale San Paolo, Civitavecchia, Roma	Civitavecchia, Roma, Italy	Data Collector	
Paolo	Bellora			Clinica Chirurgica Ospedale Maggiore della Carità - Novara	Novara, Italy	Principal Investigator; Data	
Sergio	Gentilli			Clinica Chirurgica Ospedale Maggiore della Carità - Novara	Novara, Italy	Data Collector	
Manuela	Monni			Clinica Chirurgica Ospedale Maggiore della Carità - Novara	Novara, Italy	Data Collector	
Herald	Nikaj			Clinica Chirurgica Ospedale Maggiore della Carità - Novara	Novara, Italy	Data Collector	
Nicola	Cillara			UOC Chirurgia Generale PO Santissima Trinità ASSL Cagliari	Cagliari, Italy	Principal Investigator	
Alessandro	Cannavera			UOC Chirurgia Generale PO Santissima Trinità ASSL Cagliari	Cagliari, Italy	Data Collector	
Antonello	Deserra			UOC Chirurgia Generale PO Santissima Trinità ASSL Cagliari	Cagliari, Italy	Data Collector	
Carla	Margiani			UOC Chirurgia Generale PO Santissima Trinità ASSL Cagliari	Cagliari, Italy	Data Collector	
Roberta	Cabula			UOC Chirurgia Generale PO Santissima Trinità ASSL Cagliari	Cagliari, Italy	Data Validator	
Manuela	Dettori			Oncologia Medica, PO Businco, ARNAS Cagliari	Cagliari, Italy	Data Collector	

*First Name and Middle Initial(s)	*Last Name	*Suffix (eg, Jr, III)	Academic Degrees	Institution	Location (city, state/province, country)	Role or Contribution, eg, chair, principal investigator	Group (if more than 1 Group listed in the byline) and/or Subgroup (eg, Steering Committee)
Giulia	Gramignano			SSD Oncologia, PO Nostra Signora di Bonaria San Gavino, ASL Medi	San Gavino Monreale, Medio	Data Collector	
Giovanni	Lezoche			Clinica di Chirurgia Generale e d'urgenza, Ancona Torrette	Ancona, Italy	Principal Investigator	
Monica	Ortenzi			Clinica di Chirurgia Generale e d'urgenza, Ancona Torrette	Ancona, Italy	Data Validator	
Elena S	Orlandoni			Clinica di Chirurgia Generale e d'urgenza, Ancona Torrette	Ancona, Italy	Data Collector	
Federica	Curzi			Clinica di Chirurgia Generale e d'urgenza, Ancona Torrette	Ancona, Italy	Data Collector	
Francesca	Vitali			Clinica di Chirurgia Generale e d'urgenza, Ancona Torrette	Ancona, Italy	Data Collector	
Perla	Capomagi			Clinica di Chirurgia Generale e d'urgenza, Ancona Torrette	Ancona, Italy	Data Collector	
Miriam	Palmieri			Clinica di Chirurgia Generale e d'urgenza, Ancona Torrette	Ancona, Italy	Data Collector	
Mario	Giuffrida			Clinica Chirurgica Generale - AOU Parma	Parma, Italy	Data Collector	
Paolo	Del Rio			Clinica Chirurgica Generale - AOU Parma	Parma, Italy	Data Collector	
Elena	Bonati			Clinica Chirurgica Generale - AOU Parma	Parma, Italy	Data Collector	
Tommaso	Loderer			Clinica Chirurgica Generale - AOU Parma	Parma, Italy	Data Collector	
Federico	Cozzani			Clinica Chirurgica Generale - AOU Parma	Parma, Italy	Principal Investigator	
Matteo	Rossini			Clinica Chirurgica Generale - AOU Parma	Parma, Italy	Data Collector	
Stefano	Agnesi			Clinica Chirurgica Generale - AOU Parma	Parma, Italy	Data Validator	
Gabriella T	Capolupo			UOC Chirurgia colorettale, Fondazione Policlinico Campus Bio Medi	Roma, Italy	Principal Investigator; Data	
Marco	Caricato			UOC Chirurgia colorettale, Fondazione Policlinico Campus Bio Medi	Roma, Italy	Data Collector	
Filippo	Carannante			UOC Chirurgia colorettale, Fondazione Policlinico Campus Bio Medi	Roma, Italy	Data Collector	
Gianluca	Mascianà			UOC Chirurgia colorettale, Fondazione Policlinico Campus Bio Medi	Roma, Italy	Data Collector	
Martina	Marrelli			UOC Chirurgia colorettale, Fondazione Policlinico Campus Bio Medi	Roma, Italy	Data Collector	
Valentina	Miacci			UOC Chirurgia colorettale, Fondazione Policlinico Campus Bio Medi	Roma, Italy	Data Collector	
Sara	Lauricella			UOC Chirurgia colorettale, Fondazione Policlinico Campus Bio Medi	Roma, Italy	Data Collector	
Valeria	Tonini			Ospedale Santissima Annunziata, Taranto	Taranto, Italy	Principal Investigator	
Maurizio	Cervellera			Ospedale Santissima Annunziata, Taranto	Taranto, Italy	Data Collector	
Salvatore	Pisconti			Ospedale Santissima Annunziata, Taranto	Taranto, Italy	Data Collector	
Concetta	Lozito			Ospedale Santissima Annunziata, Taranto	Taranto, Italy	Data Collector	
Juliana	Shahu			Ospedale Santissima Annunziata, Taranto	Taranto, Italy	Data Collector	
Claudia	Mongelli			Ospedale Santissima Annunziata, Taranto	Taranto, Italy	Data Collector	
Giulia	Morelli			Ospedale Santissima Annunziata, Taranto	Taranto, Italy	Data Collector	
Lodovico	Sartarelli			Ospedale Santissima Annunziata, Taranto	Taranto, Italy	Data Validator	
Giuseppe S	Sica			Policlinico Tor Vergata, Roma	Roma, Italy	Principal Investigator	
Leandro	Siragusa			Policlinico Tor Vergata, Roma	Roma, Italy	Data Collector	
Giulia	Bagaglini			Policlinico Tor Vergata, Roma	Roma, Italy	Data Collector	
Bruno	Sensi			Policlinico Tor Vergata, Roma	Roma, Italy	Data Collector	
Andrea M	Guida			Policlinico Tor Vergata, Roma	Roma, Italy	Data Collector	
Marzia	Franceschilli			Policlinico Tor Vergata, Roma	Roma, Italy	Data Collector	
Danilo	Vinci			Policlinico Tor Vergata, Roma	Roma, Italy	Data Validator	
Antonio	Taddei			Azienda Ospedaliero Universitaria Careggi, Firenze	Firenze, Italy	Principal Investigator	
Matteo	Risaliti			Azienda Ospedaliero Universitaria Careggi, Firenze	Firenze, Italy	Data Collector	
Ilenia	Bartolini			Azienda Ospedaliero Universitaria Careggi, Firenze	Firenze, Italy	Data Collector	
Maria N	Ringressi			Azienda Ospedaliero Universitaria Careggi, Firenze	Firenze, Italy	Data Collector	
Luca	Tirloni			Azienda Ospedaliero Universitaria Careggi, Firenze	Firenze, Italy	Data Validator	
Letizia	Laface			Ospedale Vittorio Emanuele III Carate Brianza	Carate Brianza, Monza-Brianza,	Principal Investigator; Data	
Emmanuele	Abate			Ospedale Vittorio Emanuele III Carate Brianza	Carate Brianza, Monza-Brianza,	Data Collector	
Massimiliano	Casati			Ospedale Vittorio Emanuele III Carate Brianza	Carate Brianza, Monza-Brianza,	Data Collector	
Pietro	Gobbi			Ospedale Vittorio Emanuele III Carate Brianza	Carate Brianza, Monza-Brianza,	Data Collector	
Enrico	Opocher			ASST Santi Paolo e Carlo, Milano	Milano, Italy	Principal Investigator	
Nicolò M	Mariani			ASST Santi Paolo e Carlo, Milano	Milano, Italy	Data Collector	

Supplemental Online Content: Nonauthor Collaborators*First name, last name, and suffix (if applicable) are required and will appear in PubMed.

*First Name and Middle Initial(s)	*Last Name	*Suffix (eg, Jr, III)	Academic Degrees	Institution	Location (city, state/province, country)	Role or Contribution, eg, chair, principal investigator	Group (if more than 1 Group listed in the byline) and/or Subgroup (eg, Steering Committee)
Andrea	Pisani Ceretti			ASST Santi Paolo e Carlo, Milano	Milano, Italy	Data Collector	
Marco	Giovenzana			ASST Santi Paolo e Carlo, Milano	Milano, Italy	Data Validator	
Beatrice	Giuliani			ASST Santi Paolo e Carlo, Milano	Milano, Italy	Data Collector	
Martina	Sironi			ASST Santi Paolo e Carlo, Milano	Milano, Italy	Data Collector	
Ugo	Grossi			II Surgery Unit, Regional Hospital Treviso, DISCOG, University of Pad	Treviso, Italy	Principal Investigator	
Giacomo	Zanus			II Surgery Unit, Regional Hospital Treviso, DISCOG, University of Pad	Treviso, Italy	Data Collector	
Giulio	Aniello Santoro			II Surgery Unit, Regional Hospital Treviso, DISCOG, University of Pad	Treviso, Italy	Data Collector	
Marco	Brizzolari			II Surgery Unit, Regional Hospital Treviso, DISCOG, University of Pad	Treviso, Italy	Data Collector	
Eugenio	De Leo			II Surgery Unit, Regional Hospital Treviso, DISCOG, University of Pad	Treviso, Italy	Data Collector	
Simone	Novello			II Surgery Unit, Regional Hospital Treviso, DISCOG, University of Pad	Treviso, Italy	Data Validator	
Krizia	Aquilino			II Surgery Unit, Regional Hospital Treviso, DISCOG, University of Pad	Treviso, Italy	Data Collector	
Francesco	Milardi			II Surgery Unit, Regional Hospital Treviso, DISCOG, University of Pad	Treviso, Italy	Data Collector	
Stefano	Olmi			Policlinico San Marco GSD, Zingonia	Zingonia, Bergamo, Italy	Principal Investigator	
Matteo	Uccelli			Policlinico San Marco GSD, Zingonia	Zingonia, Bergamo, Italy	Data Collector	
Marta	Bonaldi			Policlinico San Marco GSD, Zingonia	Zingonia, Bergamo, Italy	Data Collector	
Giovanni C	Cesana			Policlinico San Marco GSD, Zingonia	Zingonia, Bergamo, Italy	Data Collector	
Marco	Bindi			Policlinico San Marco GSD, Zingonia	Zingonia, Bergamo, Italy	Data Validator	
Raffaele	Galleano			Ospedale San Paolo Savona	Savona, Italy	Principal Investigator; Data	
Antonio	Langone			Ospedale San Paolo Savona	Savona, Italy	Data Collector	
Massimiliano	Botto			Ospedale San Paolo Savona	Savona, Italy	Data Collector	
Angelo	Franceschi			Ospedale San Paolo Savona	Savona, Italy	Data Collector	
Elena	Gambino			Ospedale San Paolo Savona	Savona, Italy	Data Collector	
Maurizio	Ronconi			Ospedale Gardone V.T. - U.O.C Chirurgia Generale	Gardone Val Trompia, Brescia, Italy	Principal Investigator	
Silvia	Casiraghi			Ospedale Gardone V.T. - U.O.C Chirurgia Generale	Gardone Val Trompia, Brescia, Italy	Data Collector	
Giovanni	Casole			Ospedale Gardone V.T. - U.O.C Chirurgia Generale	Gardone Val Trompia, Brescia, Italy	Data Collector	
Salvatore L	Ciulla			Ospedale Gardone V.T. - U.O.C Chirurgia Generale	Gardone Val Trompia, Brescia, Italy	Data Validator	
Giovanni	Terrosu			Clinica Chirurgica, Azienda Sanitaria Universitaria Friuli Centrale ASU	Udine, Italy	Principal Investigator	
Sergio	Calandra			Clinica Chirurgica, Azienda Sanitaria Universitaria Friuli Centrale ASU	Udine, Italy	Data Collector	
Edoardo	Scarpa			Clinica Chirurgica, Azienda Sanitaria Universitaria Friuli Centrale ASU	Udine, Italy	Data Collector	
Vittorio	Cherchi			Clinica Chirurgica, Azienda Sanitaria Universitaria Friuli Centrale ASU	Udine, Italy	Data Collector	
Giacomo	Calini			Clinica Chirurgica, Azienda Sanitaria Universitaria Friuli Centrale ASU	Udine, Italy	Data Collector	
Lisa	Martinuzzo			Clinica Chirurgica, Azienda Sanitaria Universitaria Friuli Centrale ASU	Udine, Italy	Data Collector	
Lucrezia	Clocchiatti			Clinica Chirurgica, Azienda Sanitaria Universitaria Friuli Centrale ASU	Udine, Italy	Data Collector	
Davide	Muschitiello			Clinica Chirurgica, Azienda Sanitaria Universitaria Friuli Centrale ASU	Udine, Italy	Data Validator	
Andrea	Romanzi			Department of General Surgery, Valduce Hospital, Como, Italy	Como, Italy	Principal Investigator; Data	
Barbara	Vignati			Department of General Surgery, Valduce Hospital, Como, Italy	Como, Italy	Data Collector	
Alberto	Vannelli			Department of General Surgery, Valduce Hospital, Como, Italy	Como, Italy	Data Collector	
Roberta	Scolaro			Department of General Surgery, Valduce Hospital, Como, Italy	Como, Italy	Data Collector	
Maria	Milanesi			Department of General Surgery, Valduce Hospital, Como, Italy	Como, Italy	Data Collector	
Fabrizio	Rossi			Department of General Surgery, Valduce Hospital, Como, Italy	Como, Italy	Data Collector	
Giuseppe	Canonico			Ospedale San Giovanni di Dio, Firenze	Firenze, Italy	Principal Investigator	
Alessandro	Anastasi			Ospedale San Giovanni di Dio, Firenze	Firenze, Italy	Data Collector	
Tommaso	Nelli			Ospedale San Giovanni di Dio, Firenze	Firenze, Italy	Data Collector	
Marco	Barlettai			Ospedale San Giovanni di Dio, Firenze	Firenze, Italy	Data Collector	
Riccardo	Fratarcangeli			Ospedale San Giovanni di Dio, Firenze	Firenze, Italy	Data Collector	
Carmela	Di Martino			Ospedale San Giovanni di Dio, Firenze	Firenze, Italy	Data Collector	
Andrea	Damigella			Ospedale San Giovanni di Dio, Firenze	Firenze, Italy	Data Collector	

*First Name and Middle Initial(s)	*Last Name	*Suffix (eg, Jr, III)	Academic Degrees	Institution	Location (city, state/province, country)	Role or Contribution, eg, chair, principal investigator	Group (if more than 1 Group listed in the byline) and/or Subgroup (eg, Steering Committee)
Elvira	Adinolfi			Ospedale San Giovanni di Dio, Firenze	Firenze, Italy	Data Validator	
Arianna	Birindelli			UOC Chirurgia - Ospedale di Esine (BS) - ASST Valcamonica - Italy	Esine, Brescia, Italy	Principal Investigator; Data	
Lucio	Taglietti			UOC Chirurgia - Ospedale di Esine (BS) - ASST Valcamonica - Italy	Esine, Brescia, Italy	Data Collector	
Sara E	Dester			UOC Chirurgia - Ospedale di Esine (BS) - ASST Valcamonica - Italy	Esine, Brescia, Italy	Data Collector	
Francesco	Fleres			AOU G. Martino Policlinico di Messina, Department of General and	Messina, Italy	Principal Investigator	
Eugenio	Cucinotta			AOU G. Martino Policlinico di Messina, Department of General and	Messina, Italy	Data Collector	
Francesca	Viscosi			AOU G. Martino Policlinico di Messina, Department of General and	Messina, Italy	Data Collector	
Antonio	Biondo Santino			AOU G. Martino Policlinico di Messina, Department of General and	Messina, Italy	Data Collector	
Giorgio	Badessi			AOU G. Martino Policlinico di Messina, Department of General and	Messina, Italy	Data Collector	
Nivia	Catarsini			AOU G. Martino Policlinico di Messina, Department of General and	Messina, Italy	Data Collector	
Carmelo	Mazzeo			AOU G. Martino Policlinico di Messina, Department of General and	Messina, Italy	Data Validator	
Daniela	Rega			Colorectal Surgical Oncology, Department of Abdominal Oncology,	Napoli, Italy	Principal Investigator	
Paolo	Delrio			Colorectal Surgical Oncology, Department of Abdominal Oncology,	Napoli, Italy	Data Collector	
Carmela	Cervone			Colorectal Surgical Oncology, Department of Abdominal Oncology,	Napoli, Italy	Data Collector	
Alessia	Aversano			Colorectal Surgical Oncology, Department of Abdominal Oncology,	Napoli, Italy	Data Collector	
Silvia	De Franciscis			Colorectal Surgical Oncology, Department of Abdominal Oncology,	Napoli, Italy	Data Collector	
Massimiliano	Di Marzo			Colorectal Surgical Oncology, Department of Abdominal Oncology,	Napoli, Italy	Data Collector	
Bruno	Marra			Colorectal Surgical Oncology, Department of Abdominal Oncology,	Napoli, Italy	Data Collector	
Ugo	Pace			Colorectal Surgical Oncology, Department of Abdominal Oncology,	Napoli, Italy	Data Validator	
Antonio	Amato			SC Chirurgia Generale Imperia	Imperia, Italy	Principal Investigator	
Paola	Batistotti			SC Chirurgia Generale Imperia	Imperia, Italy	Data Collector	
Elisa	Mina			SC Chirurgia Generale Imperia	Imperia, Italy	Data Collector	
Alberto	Serventi			SC Chirurgia Generale Imperia	Imperia, Italy	Data Validator	
Pierfrancesco	Lapolla			Policlinico Umberto I Sapienza Università di Roma	Roma, Italy	Principal Investigator	
Andrea	Mingoli			Policlinico Umberto I Sapienza Università di Roma	Roma, Italy	Data Collector	
Paolo	Sapienza			Policlinico Umberto I Sapienza Università di Roma	Roma, Italy	Data Collector	
Gioia	Brachini			Policlinico Umberto I Sapienza Università di Roma	Roma, Italy	Data Collector	
Bruno	Cirillo			Policlinico Umberto I Sapienza Università di Roma	Roma, Italy	Data Collector	
Enrico	Fiori			Policlinico Umberto I Sapienza Università di Roma	Roma, Italy	Data Collector	
Daniele	Crocetti			Policlinico Umberto I Sapienza Università di Roma	Roma, Italy	Data Collector	
Ilaria	Clementi			Policlinico Umberto I Sapienza Università di Roma	Roma, Italy	Data Validator	
Gennaro	Martines			Chirurgia Generale "M.Rubino" Azienda Ospedaliero Universitaria P	Bari, Italy	Principal Investigator	
Arcangelo	Picciariello			Chirurgia Generale "M.Rubino" Azienda Ospedaliero Universitaria P	Bari, Italy	Data Collector	
Giovanni	Tomasicchio			Chirurgia Generale "M.Rubino" Azienda Ospedaliero Universitaria P	Bari, Italy	Data Validator	
Rigers	Dibra			Chirurgia Generale "M.Rubino" Azienda Ospedaliero Universitaria P	Bari, Italy	Data Collector	
Giuseppe	Trigiante			Chirurgia Generale "M.Rubino" Azienda Ospedaliero Universitaria P	Bari, Italy	Data Collector	
Marcella	Rinaldi			Chirurgia Generale "M.Rubino" Azienda Ospedaliero Universitaria P	Bari, Italy	Data Collector	
Giuliano	Lantone			Chirurgia Generale "M.Rubino" Azienda Ospedaliero Universitaria P	Bari, Italy	Data Collector	
Alberto	Porcu			Azienda Ospedaliero Universitaria di Sassari, Italia	Sassari, Italy	Principal Investigator	
Teresa	Perra			Azienda Ospedaliero Universitaria di Sassari, Italia	Sassari, Italy	Data Collector	
Antonio M	Scanu			Azienda Ospedaliero Universitaria di Sassari, Italia	Sassari, Italy	Data Validator	
Claudio F	Feo			Azienda Ospedaliero Universitaria di Sassari, Italia	Sassari, Italy	Data Collector	
Alessandro	Fancellu			Azienda Ospedaliero Universitaria di Sassari, Italia	Sassari, Italy	Data Collector	
Maria L	Cossu			Azienda Ospedaliero Universitaria di Sassari, Italia	Sassari, Italy	Data Collector	
Giorgio C	Ginesu			Azienda Ospedaliero Universitaria di Sassari, Italia	Sassari, Italy	Data Collector	
Alberto	Patriti			AO Ospedali Riuniti Marche Nord	Pesaro, Pesaro-Urbino, Italy	Principal Investigator	
Diego	Coletta			AO Ospedali Riuniti Marche Nord	Pesaro, Pesaro-Urbino, Italy	Data Validator	
Filippo	Petrelli			AO Ospedali Riuniti Marche Nord	Pesaro, Pesaro-Urbino, Italy	Data Collector	

Supplemental Online Content: Nonauthor Collaborators*First name, last name, and suffix (if applicable) are required and will appear in PubMed.

*First Name and Middle Initial(s)	*Last Name	*Suffix (eg, Jr, III)	Academic Degrees	Institution	Location (city, state/province, country)	Role or Contribution, eg, chair, principal investigator	Group (if more than 1 Group listed in the byline) and/or Subgroup (eg, Steering Committee)
Paola A	Greco			AO Ospedali Riuniti Marche Nord	Pesaro, Pesaro-Urbino, Italy	Data Collector	
Claudia	Spadoni			AO Ospedali Riuniti Marche Nord	Pesaro, Pesaro-Urbino, Italy	Data Collector	
Giovanna	Cassiani			AO Ospedali Riuniti Marche Nord	Pesaro, Pesaro-Urbino, Italy	Data Collector	
Federica	Bianchini			AO Ospedali Riuniti Marche Nord	Pesaro, Pesaro-Urbino, Italy	Data Collector	
Marco	Arganini			Ospedale Unico della Versilia - Azienda Usl Toscana Nord-ovest	Camaiore, Lucca, Italy	Principal Investigator	
Matteo	Bianchini			Ospedale Unico della Versilia - Azienda Usl Toscana Nord-ovest	Camaiore, Lucca, Italy	Data Validator	
Bruno	Perotti			Ospedale Unico della Versilia - Azienda Usl Toscana Nord-ovest	Camaiore, Lucca, Italy	Data Collector	
Matteo	Palmeri			Ospedale Unico della Versilia - Azienda Usl Toscana Nord-ovest	Camaiore, Lucca, Italy	Data Collector	
Stefano	Scabini			Unità Operativa Chirurgia Generale ad Indirizzo Oncologico - IRCCS	Genova, Italy	Principal Investigator; Data	
Selene	Deiana			Unità Operativa Chirurgia Generale ad Indirizzo Oncologico - IRCCS	Genova, Italy	Data Collector	
Giacomo	Carganico			Unità Operativa Chirurgia Generale ad Indirizzo Oncologico - IRCCS	Genova, Italy	Data Collector	
Davide	Pertile			Unità Operativa Chirurgia Generale ad Indirizzo Oncologico - IRCCS	Genova, Italy	Data Collector	
Domenico	Soriero			Unità Operativa Chirurgia Generale ad Indirizzo Oncologico - IRCCS	Genova, Italy	Data Collector	
Emanuela	Fioravanti			Unità Operativa Chirurgia Generale ad Indirizzo Oncologico - IRCCS	Genova, Italy	Data Collector	
Beatrice	Sperotto			Unità Operativa Chirurgia Generale ad Indirizzo Oncologico - IRCCS	Genova, Italy	Data Collector	
Bruno	Nardo			U.O.C. di Chirurgia Generale "Falcone" - Azienda Ospedaliera di Cos	Cosenza, Italy	Principal Investigator	
Daniele	Paglione			U.O.C. di Chirurgia Generale "Falcone" - Azienda Ospedaliera di Cos	Cosenza, Italy	Data Validator	
Veronica	Crocco			U.O.C. di Chirurgia Generale "Falcone" - Azienda Ospedaliera di Cos	Cosenza, Italy	Data Collector	
Marco	Doni			U.O.C. di Chirurgia Generale "Falcone" - Azienda Ospedaliera di Cos	Cosenza, Italy	Data Collector	
Mariasara	Osso			U.O.C. di Chirurgia Generale "Falcone" - Azienda Ospedaliera di Cos	Cosenza, Italy	Data Collector	
Roberto	Perri			U.O.C. di Chirurgia Generale "Falcone" - Azienda Ospedaliera di Cos	Cosenza, Italy	Data Collector	
Gianluca M	Sampietro			Division of Surgery, Rho Memorial Hospital - ASST Rhodense - Rho,	Rho, Milano, Italy	Principal Investigator	
Carlo	Corbellini			Division of Surgery, Rho Memorial Hospital - ASST Rhodense - Rho,	Rho, Milano, Italy	Data Validator	
Leonardo	Lorusso			Division of Surgery, Rho Memorial Hospital - ASST Rhodense - Rho,	Rho, Milano, Italy	Data Collector	
Carlo A	Manzo			Division of Surgery, Rho Memorial Hospital - ASST Rhodense - Rho,	Rho, Milano, Italy	Data Collector	
Maria	Cigognini			Division of Surgery, Rho Memorial Hospital - ASST Rhodense - Rho,	Rho, Milano, Italy	Data Collector	
Caterina	Baldi			Division of Surgery, Rho Memorial Hospital - ASST Rhodense - Rho,	Rho, Milano, Italy	Data Collector	
Giuseppe	Palomba			AOU Federico II di Napoli - UOC chirurgia endoscopica	Napoli, Italy	Principal Investigator	
Giovanni	Aprea			AOU Federico II di Napoli - UOC chirurgia endoscopica	Napoli, Italy	Data Collector	
Marianna	Capuano			AOU Federico II di Napoli - UOC chirurgia endoscopica	Napoli, Italy	Data Validator	
Raffaele	Basile			AOU Federico II di Napoli - UOC chirurgia endoscopica	Napoli, Italy	Data Collector	
Roberta	Tutino			Chirurgia 1 - Azienda ULSS2 Marca Trevigiana - Ospedale Regionale	Treviso, Italy	Principal Investigator; Data	
Marco	Massani			Chirurgia 1 - Azienda ULSS2 Marca Trevigiana - Ospedale Regionale	Treviso, Italy	Data Collector	
Laura	Marinelli			Chirurgia 1 - Azienda ULSS2 Marca Trevigiana - Ospedale Regionale	Treviso, Italy	Data Collector	
Nicola	Canitano			Chirurgia 1 - Azienda ULSS2 Marca Trevigiana - Ospedale Regionale	Treviso, Italy	Data Collector	
Tiziana	Pilia			Policlinico di Monserrato, Chirurgia d'urgenza, Cagliari	Cagliari, Italy	Principal Investigator	
Mauro	Podda			Policlinico di Monserrato, Chirurgia d'urgenza, Cagliari	Cagliari, Italy	Data Collector	
Adolfo	Pisanu			Policlinico di Monserrato, Chirurgia d'urgenza, Cagliari	Cagliari, Italy	Data Collector	
Valentina	Murzi			Policlinico di Monserrato, Chirurgia d'urgenza, Cagliari	Cagliari, Italy	Data Collector	
Silvia	Incani			Policlinico di Monserrato, Chirurgia d'urgenza, Cagliari	Cagliari, Italy	Data Validator	
Federica	Frongia			Policlinico di Monserrato, Chirurgia d'urgenza, Cagliari	Cagliari, Italy	Data Collector	
Giuseppe	Esposito			Policlinico di Monserrato, Chirurgia d'urgenza, Cagliari	Cagliari, Italy	Data Collector	
Gaetano	Luglio			Azienda Ospedaliera Universitaria Federico II	Napoli, Italy	Principal Investigator	
Francesca P	Tropeano			Azienda Ospedaliera Universitaria Federico II	Napoli, Italy	Data Collector	
Gianluca	Pagano			Azienda Ospedaliera Universitaria Federico II	Napoli, Italy	Data Collector	
Eduardo	Spina			Azienda Ospedaliera Universitaria Federico II	Napoli, Italy	Data Collector	
Giuseppe	De Simone			Azienda Ospedaliera Universitaria Federico II	Napoli, Italy	Data Collector	
Michele	Cricrì			Azienda Ospedaliera Universitaria Federico II	Napoli, Italy	Data Validator	

*First Name and Middle Initial(s)	*Last Name	*Suffix (eg, Jr, III)	Academic Degrees	Institution	Location (city, state/province, country)	Role or Contribution, eg, chair, principal investigator	Group (if more than 1 Group listed in the byline) and/or Subgroup (eg, Steering Committee)
Fausto	Catena			Chirurgia Generale e d'Urgenza, Ospedale Bufalini di Cesena, AUSL	Cesena, Forlì-Cesena, Italy	Principal Investigator	
Carlo	Vallicelli			Chirurgia Generale e d'Urgenza, Ospedale Bufalini di Cesena, AUSL	Cesena, Forlì-Cesena, Italy	Data Validator	
Nicola	Zanini			Chirurgia Generale e d'Urgenza, Ospedale Bufalini di Cesena, AUSL	Cesena, Forlì-Cesena, Italy	Data Collector	
Diana	Ronconi			Chirurgia Generale e d'Urgenza, Ospedale Bufalini di Cesena, AUSL	Cesena, Forlì-Cesena, Italy	Data Collector	
Francesco	Favi			Chirurgia Generale e d'Urgenza, Ospedale Bufalini di Cesena, AUSL	Cesena, Forlì-Cesena, Italy	Data Collector	
Carlo	Mazzucchelli			Chirurgia Generale e d'Urgenza, Ospedale Bufalini di Cesena, AUSL	Cesena, Forlì-Cesena, Italy	Data Collector	
Girolamo	Convertini			Chirurgia Generale e d'Urgenza, Ospedale Bufalini di Cesena, AUSL	Cesena, Forlì-Cesena, Italy	Data Collector	
Leonardo	Vincenti			Chirurgia Generale Ospedaliera, Policlinico di Bari	Bari, Italy	Principal Investigator	
Valeria	Andriola			Chirurgia Generale Ospedaliera, Policlinico di Bari	Bari, Italy	Data Collector	
Cinzia	Bizzoca			Chirurgia Generale Ospedaliera, Policlinico di Bari	Bari, Italy	Data Validator	
Carlo V	Feo			Azienda Unità Sanitaria Locale di Ferrara, Università di Ferrara	Ferrara, Italy	Principal Investigator	
Nicolò	Fabbri			Azienda Unità Sanitaria Locale di Ferrara, Università di Ferrara	Ferrara, Italy	Data Validator	
Marta	Fazzin			Azienda Unità Sanitaria Locale di Ferrara, Università di Ferrara	Ferrara, Italy	Data Collector	
Antonio	Pesce			Azienda Unità Sanitaria Locale di Ferrara, Università di Ferrara	Ferrara, Italy	Data Collector	
Silvia	Gennari			Azienda Unità Sanitaria Locale di Ferrara, Università di Ferrara	Ferrara, Italy	Data Collector	
Marco	Torchiaro			Azienda Unità Sanitaria Locale di Ferrara, Università di Ferrara	Ferrara, Italy	Data Collector	
Silvia	Severi			Azienda Unità Sanitaria Locale di Ferrara, Università di Ferrara	Ferrara, Italy	Data Collector	
Alice	Frontali			General Surgery Unit, ASST Vimercate, Vimercate, Italy	Vimercate, Monza-Brianza, Italy	Principal Investigator; Data	
Greta	Bracchetti			General Surgery Unit, ASST Vimercate, Vimercate, Italy	Vimercate, Monza-Brianza, Italy	Data Collector	
Stefano	Granieri			General Surgery Unit, ASST Vimercate, Vimercate, Italy	Vimercate, Monza-Brianza, Italy	Data Collector	
Christian	Cotsoglou			General Surgery Unit, ASST Vimercate, Vimercate, Italy	Vimercate, Monza-Brianza, Italy	Data Collector	
Massimo	Carlini			UOC Chirurgia Generale, Ospedale Sant'Eugenio, Roma, Italia	Roma, Italy	Principal Investigator	
Giorgio	Lisi			UOC Chirurgia Generale, Ospedale Sant'Eugenio, Roma, Italia	Roma, Italy	Data Collector	
Domenico	Spoletini			UOC Chirurgia Generale, Ospedale Sant'Eugenio, Roma, Italia	Roma, Italy	Data Collector	
Maria R	Mastrangeli			UOC Chirurgia Generale, Ospedale Sant'Eugenio, Roma, Italia	Roma, Italy	Data Collector	
Michela	Campanelli			UOC Chirurgia Generale, Ospedale Sant'Eugenio, Roma, Italia	Roma, Italy	Data Validator	
Michele	Manigrasso			Department of Clinical Medicine and Surgery, "Federico II"	Napoli, Italy	Principal Investigator	
Marco	Milone			Department of Clinical Medicine and Surgery, "Federico II"	Napoli, Italy	Data Collector	
Giovanni D	De Palma			Department of Clinical Medicine and Surgery, "Federico II"	Napoli, Italy	Data Collector	
Sara	Vertaldi			Department of Clinical Medicine and Surgery, "Federico II"	Napoli, Italy	Data Collector	
Alessia	Chini			Department of Clinical Medicine and Surgery, "Federico II"	Napoli, Italy	Data Collector	
Francesco	Maione			Department of Clinical Medicine and Surgery, "Federico II"	Napoli, Italy	Data Validator	
Alessandra	Marello			Department of Clinical Medicine and Surgery, "Federico II"	Napoli, Italy	Data Collector	
Francesco	Selvaggi			Università della Campania Luigi Vanvitelli, Napoli	Napoli, Italy	Data Collector	
Guido	Sciaudone			Università della Campania Luigi Vanvitelli, Napoli	Napoli, Italy	Data Collector	
Lucio	Selvaggi			Università della Campania Luigi Vanvitelli, Napoli	Napoli, Italy	Data Collector	
Francesco	Menegon Tasselli			Università della Campania Luigi Vanvitelli, Napoli	Napoli, Italy	Data Collector	
Giacomo	Fuschillo			Università della Campania Luigi Vanvitelli, Napoli	Napoli, Italy	Data Collector	
Lidia	Oddis			Università della Campania Luigi Vanvitelli, Napoli	Napoli, Italy	Data Collector	
Michela	Campanelli			UOSD Chirurgia d'urgenza Tor Vergata	Roma, Italy	Principal Investigator; Data	
Simona	Grande			UOSD Chirurgia d'urgenza Tor Vergata	Roma, Italy	Data Collector	
Michele	Grande			UOSD Chirurgia d'urgenza Tor Vergata	Roma, Italy	Data Collector	
Simona	Ascanelli			UO Chirurgia 2 Azienda Ospedaliero-Universitaria Ferrara	Ferrara, Italy	Principal Investigator; Data	
Laura	Chimisso			UO Chirurgia 2 Azienda Ospedaliero-Universitaria Ferrara	Ferrara, Italy	Data Collector	
Filippo	Aisoni			UO Chirurgia 2 Azienda Ospedaliero-Universitaria Ferrara	Ferrara, Italy	Data Collector	

Supplemental Online Content: Nonauthor Collaborators*First name, last name, and suffix (if applicable) are required and will appear in PubMed.

*First Name and Middle Initial(s)	*Last Name	*Suffix (eg, Jr, III)	Academic Degrees	Institution	Location (city, state/province, country)	Role or Contribution, eg, chair, principal investigator	Group (if more than 1 Group listed in the byline) and/or Subgroup (eg, Steering Committee)
Eleonora	Rossin			UO Chirurgia 2 Azienda Ospedaliero-Universitaria Ferrara	Ferrara, Italy	Data Collector	
Francesco	Pepe			UO Chirurgia 2 Azienda Ospedaliero-Universitaria Ferrara	Ferrara, Italy	Data Collector	
Francesco	Marchetti			UO Chirurgia 2 Azienda Ospedaliero-Universitaria Ferrara	Ferrara, Italy	Data Collector	
Biagio	Picardi			Chirurgia Generale e d'Urgenza Ospedale San Filippo Neri ASL	Roma, Italy	Principal Investigator; Data	
Stefano	Rossi			Chirurgia Generale e d'Urgenza Ospedale San Filippo Neri ASL	Roma, Italy	Data Collector	
Simone	Rossi Del Monte			Chirurgia Generale e d'Urgenza Ospedale San Filippo Neri ASL	Roma, Italy	Data Collector	
Matteo	Picarelli			Chirurgia Generale e d'Urgenza Ospedale San Filippo Neri ASL	Roma, Italy	Data Collector	
Irnerio A	Muttillo			Chirurgia Generale e d'Urgenza Ospedale San Filippo Neri ASL	Roma, Italy	Data Collector	
Carlo	Ratto			Fondazione Policlinico Universitario Agostino Gemelli, IRCCS,	Roma, Italy	Principal Investigator	
Angelo A	Marra			Fondazione Policlinico Universitario Agostino Gemelli, IRCCS,	Roma, Italy	Data Collector	
Angelo	Parello			Fondazione Policlinico Universitario Agostino Gemelli, IRCCS,	Roma, Italy	Data Validator	
Francesco	Litta			Fondazione Policlinico Universitario Agostino Gemelli, IRCCS,	Roma, Italy	Data Collector	
Paola	Campennì			Fondazione Policlinico Universitario Agostino Gemelli, IRCCS,	Roma, Italy	Data Collector	
Veronica	De Simone			Fondazione Policlinico Universitario Agostino Gemelli, IRCCS,	Roma, Italy	Data Collector	
Francesco	Pata			Department of Surgery, Nicola Giannettasio Hospital, Corigliano-Ro	Roma, Italy	Data Collector	
Cristiana	Riboni			EOC Ospedale Regionale di Lugano, Lugano, Switzerland	Lugano, Switzerland	Data Collector	
Emanuele	Rausa			Unit of Hereditary Digestive Tumors, Fondazione IRCCS-National	Milano, Italy	Data Collector	
Valerio	Celentano			Chelsea and Westminster Hospital NHS Foundation Trust, London,	London, UK	Data Collector	
