# Supplementary material for: Colorectal Cancer Stage at Diagnosis Before vs During the COVID-19 Pandemic in Italy
Source: JAMA Netw Open. 2022 Nov 21;5(11):e2243119. doi: 10.1001/jamanetworkopen.2022.43119 (PMC9679872; doi:10.1001/jamanetworkopen.2022.43119)
Supplement: Supplement 3. — Data Sharing Statement [file jamanetwopen-e2243119-s003.pdf]

Data Sharing Statement

Rottoli. Colorectal Cancer Stage at Diagnosis Before vs During the COVID-19 Pandemic in Italy. *JAMA Netw Open*. Published November 21, 2022.
doi:10.1001/jamanetworkopen.2022.43119

Data
Data available: Yes
Data types: Deidentified participant data, Data dictionary
How to access data: matteo.rottoli2@unibo.it
When available: With publication

Supporting Documents
Document types: None

Additional Information
Who can access the data: N/A
Types of analyses: N/A
Mechanisms of data availability: N/A
